# Supplementary material for: Convergent evolution in angiosperms adapted to cold climates
Source: Plant Commun. 2025 Jan 23;6(2):101258. doi: 10.1016/j.xplc.2025.101258 (PMC11897497; doi:10.1016/j.xplc.2025.101258)
Supplement: Document S1. Figures S1 and S2 [file mmc1.pdf]

Plant Communications, Volume *6*

Supplemental information

Convergent evolution in angiosperms adapted to cold climates

Shuo Wang, Jing Li, Ping Yu, Liangyu Guo, Junhui Zhou, Jian Yang, and Wenwu Wu

Supplementary materials

Cold climate-driven convergent evolution among angiosperms

Shuo Wang[1,6], Jing Li[1,6], Ping Yu[2,3,6], Liangyu Guo[1], Junhui Zhou[2,3], Jian Yang[2,3*], Wenwu Wu[1,4,5*]

[1]State Key Laboratory of Subtropical Silviculture, College of Forestry and Biotechnology, Zhejiang A&F University; Hangzhou 311300, China.

[2]State Key Laboratory for Quality Ensurance and Sustainable Use of Dao-di Herbs, National Resource Center for Chinese Materia Medica, China Academy of Chinese Medical Sciences; Beijing 100700, China

[3]Evaluation and Research Center of Daodi Herbs of Jiangxi Province, Ganjiang New District, 330000, China

[4]Zhejiang International Science and Technology Cooperation Base for Plant Germplasm Resources Conservation and Utilization, Zhejiang A&F University; Hangzhou 311300, China

[5]Provincial Key Laboratory for Non-wood Forest and Quality Control and Utilization of Its Products, Zhejiang A&F University, Hangzhou 311300, China

[6]These authors contributed equally to this work

***Email:** yangchem2012@163.com; wwwu@zafu.edu.cn

This file includes:

Figure S1 to S2

References

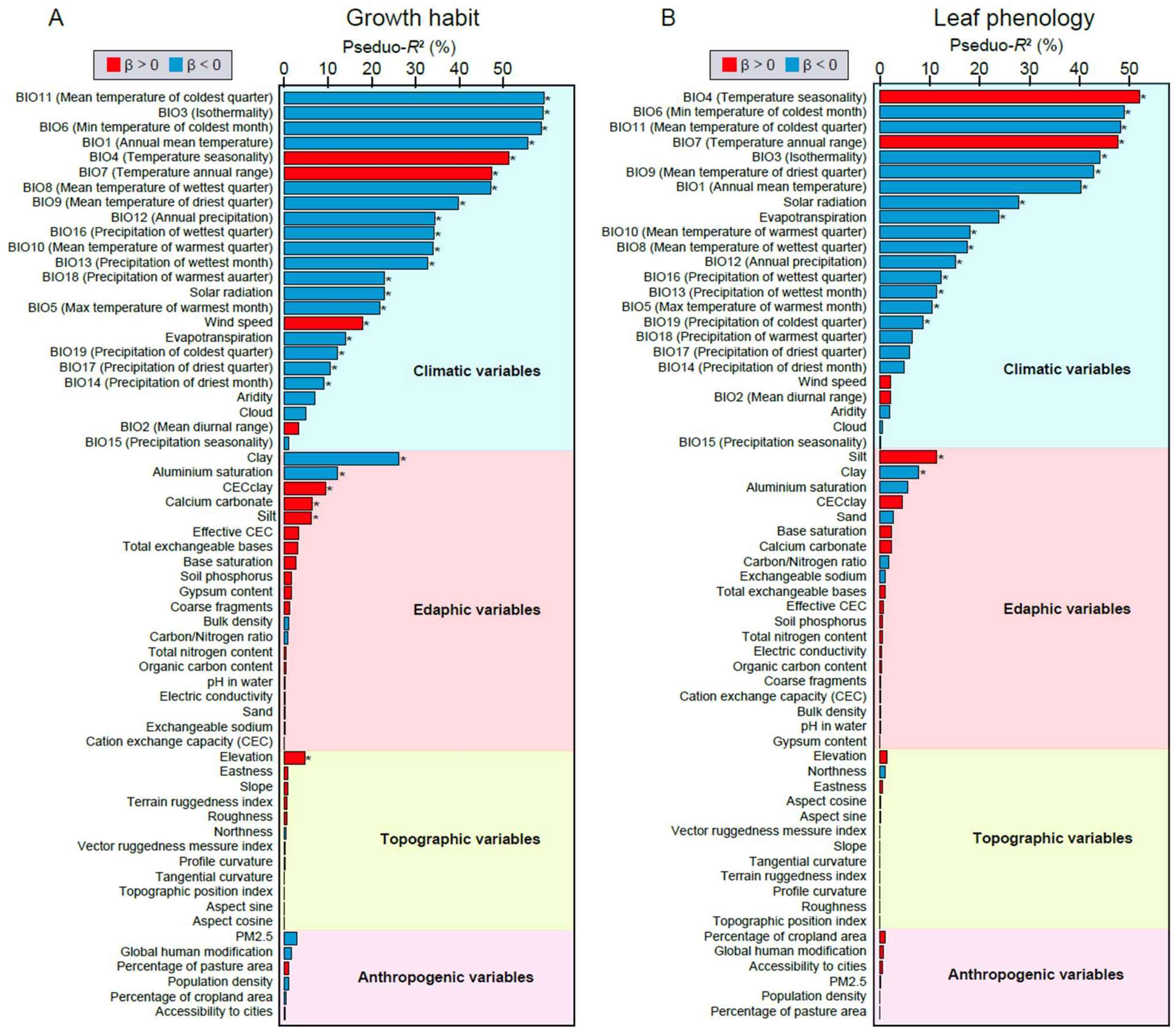

Supplementary Figure 1. Associations between geoenvironmental variables and plant traits. Beta regression was used to assess the association between each geoenvironmental variable (n = 62) and the frequency of herbaceous (**A**) and deciduous plants (**B**) across global ecoregions. The bar height represents the explained variance (pseudo-R^2), with positive effects ($\beta > 0$) indicated in red and negative effects ($\beta < 0$) in blue. To address potential spatial autocorrelation, a modified t-test was employed to assess their significance, followed by multiple testing correction using the Benjamini-Hochberg method. Statistically significant associations ($P_{adj} < 0.01$) are denoted by asterisks (*). The variables analyzed were sourced from public databases, including 19 bioclimatic variables (BIO1-BIO19) (CHELSA: chelsa-climate.org) (Karger et al., 2017), cloud cover (EarthEnv: www.earthenv.org/cloud), evapotranspiration and aridity (Zomer et al., 2022), cropland and pasture area (EarthStat: www.earthstat.org), accessibility to cities (Resource Watch: resourcewatch.org), and PM2.5 (SEDAC: sedac.ciesin.columbia.edu). The sources of other geoenvironmental variables have been described in our previous study (Wu et al., 2024).

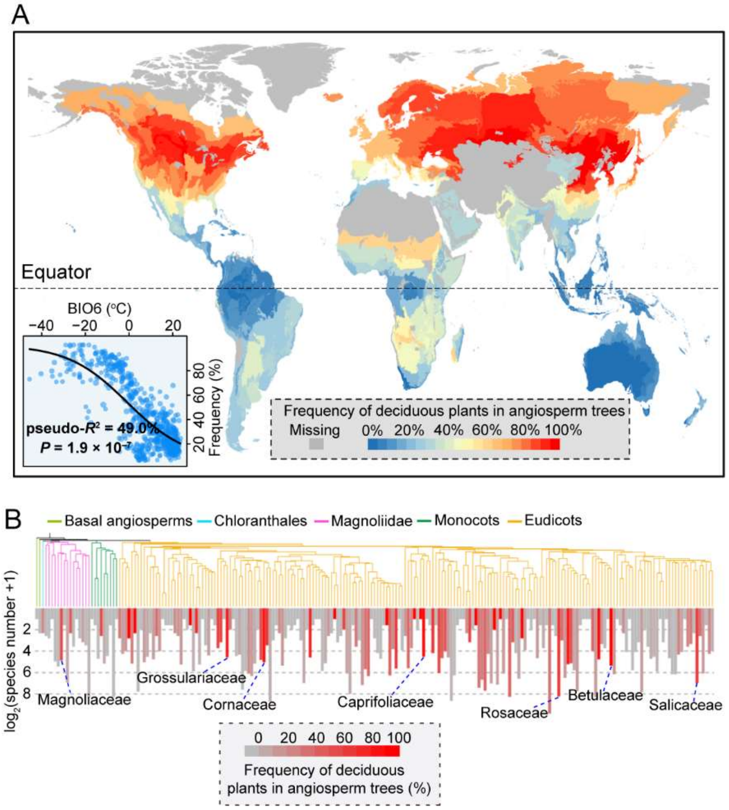

Supplementary Figure 2. Convergent evolution of leaf deciduousness in freezing temperatures. (**A**) An ecoregion-level map of the frequency of deciduous woody species. The frequency of deciduous woody plants at ecoregion level is defined as their proportion relative to the total number of deciduous and evergreen woody species collected within each ecoregion. Among the 55,557 woody angiosperm species (Fig 3A), many lacked deciduous or evergreen annotations. We supplemented these data with publicly available information (Weigelt et al., 2020; Zanne et al., 2014), yielding 2,086 deciduous and 6,757 evergreen woody plant species. At the lower left corner, we used the same method as Figure 3A to evaluate the association of deciduous frequency and BIO6.

(**B**) Independent evolution of leaf deciduousness across angiosperm woody species. Angiosperm families containing woody species were used to construct this phylogenetic tree. Below the angiosperm family tree, a bar graph shows the log2-transformed number of deciduous woody species per family in frost zones. The bars are shaded to indicate the proportion of deciduous woody species within each family that can inhabit frost zones, with darker shades representing higher proportions. A woody species was considered capable of inhabiting frost zones if it had at least five valid geographical records in these areas, and only ecoregions with twenty or more woody species were included in the analysis.

References:

Karger, D.N., Conrad, O., Bohner, J., Kawohl, T., Kreft, H., Soria-Auza, R.W., Zimmermann, N.E., Linder, H.P., and Kessler, M. (2017). Climatologies at high resolution for the earth's land surface areas. Sci Data 4:170122.

Weigelt, P., König, C., and Kreft, H. (2020). GIFT - A Global Inventory of Floras and Traits for macroecology and biogeography. J Biogeogr 47:16-43.

Wu, W., Guo, L., Yin, L., Cai, B., Li, J., Li, X., Yang, J., Zhou, H., Tao, Z., and Li, Y. (2024). Genomic convergence in terrestrial root plants through tandem duplication in response to soil microbial pressures. Cell Rep 43:114786.

Zanne, A.E., Tank, D.C., Cornwell, W.K., Eastman, J.M., Smith, S.A., FitzJohn, R.G., McGlinn, D.J., O'Meara, B.C., Moles, A.T., and Reich, P.B., et al. (2014). Three keys to the radiation of angiosperms into freezing environments. Nature 506:89-92.

Zomer, R.J., Xu, J., and Trabucco, A. (2022). Version 3 of the Global Aridity Index and Potential Evapotranspiration Database. Sci Data 9:409.
