# Supplementary material for: Convergent evolution in angiosperms adapted to cold climates
Source: Plant Commun. 2025 Jan 23;6(2):101258. doi: 10.1016/j.xplc.2025.101258 (PMC11897497; doi:10.1016/j.xplc.2025.101258)
Supplement: Document S2. Article plus supplemental information [file mmc2.pdf]

Plant Communications
Review article

CellPress
Partner Journal

Convergent evolution in angiosperms adapted to cold climates

Shuo Wang[1,6], Jing Li[1,6], Ping Yu[2,3,6], Liangyu Guo[1], Junhui Zhou[2,3], Jian Yang[2,3,*] and Wenwu Wu[1,4,5,*]

[1]National Key Laboratory for Development and Utilization of Forest Food Resources, Zhejiang A&F University, Hangzhou 311300, China

[2]State Key Laboratory for Quality Ensurance and Sustainable Use of Dao-di Herbs, National Resource Center for Chinese Materia Medica, China Academy of Chinese Medical Sciences, Beijing 100700, China

[3]Evaluation and Research Center of Daodi Herbs of Jiangxi Province, Ganjiang New District 330000, China

[4]Zhejiang International Science and Technology Cooperation Base for Plant Germplasm Resources Conservation and Utilization, Zhejiang A&F University, Hangzhou 311300, China

[5]Provincial Key Laboratory for Non-wood Forest and Quality Control and Utilization of Its Products, Zhejiang A&F University, Hangzhou 311300, China

[6]These authors contributed equally to this article.

***Correspondence: Jian Yang (yangchem2012@163.com), Wenwu Wu (wwwu@zafu.edu.cn)**

https://doi.org/10.1016/j.xplc.2025.101258

ABSTRACT

Convergent and parallel evolution occur more frequently than previously thought. Here, we focus on the evolutionary adaptations of angiosperms at sub-zero temperatures. We begin by introducing the history of research on convergent and parallel evolution, defining all independent similarities as convergent evolution. Our analysis reveals that frost zones (periodic or constant), which cover 49.1% of Earth's land surface, host 137 angiosperm families, with over 90% of their species thriving in these regions. In this context, we revisit the global biogeography and evolutionary trajectories of plant traits, such as herbaceous form and deciduous leaves, that are thought to be evasion strategies for frost adaptation. At the physiological and molecular levels, many angiosperms have independently evolved cold acclimation mechanisms through multiple pathways in addition to the well-characterized C-repeat binding factor/dehydration-responsive element binding protein 1 (CBF/DREB1) regulatory pathway. These convergent adaptations have occurred across various molecular levels, including amino acid substitutions and changes in gene duplication and expression within the same or similar functional pathways; however, identical amino acid changes are rare. Our results also highlight the prevalence of polyploidy in frost zones and the occurrence of paleopolyploidization events during global cooling. These patterns suggest repeated evolution in cold climates. Finally, we discuss plant domestication and predict climate zone shifts due to global warming and their effects on plant migration and *in situ* adaptation. Overall, the integration of ecological and molecular perspectives is essential for understanding and forecasting plant responses to climate change.

Key words: convergent evolution, angiosperm, freezing tolerance, polyploidization, tandem duplication, global warming

Wang S., Li J., Yu P., Guo L., Zhou J., Yang J., and Wu W. (2025). Convergent evolution in angiosperms adapted to cold climates. Plant Comm. **6**, 101258.

INTRODUCTION

In 1859, Charles Darwin published "On the Origin of Species," laying the foundation of evolutionary theory (Darwin, 1859). Since then, evolution has often been depicted through the lens of adaptive radiation (Schluter, 1996; Rainey and Travisano, 1998). By contrast, parallel and convergent evolution show that similar traits or functions can emerge under comparable environmental pressures, suggesting that biological evolution may be repeatable and predictable (Bolnick et al., 2018; James et al., 2023). Although Darwin acknowledged these occurrences, he considered them relatively minor, noting, "...Notwithstanding this general parallelism in the conditions of the Old and New Worlds, how widely different are their living productions!" (Darwin, 1859) and further stating, "With respect to 'convergence' I daresay, it has occurred, but I should think on a very limited scale..." (Darwin, 1993). Similarly, Stephen Jay Gould expressed skepticism about the prevalence and predictability of convergent and parallel evolution, highlighting the role of historical contingency. In his book "Wonderful Life,"

© 2025 The Author(s). Published by Elsevier Inc. on behalf of CAS Center for Excellence in Molecular Plant Sciences, Chinese Academy of Sciences, and Chinese Society for Plant Biology. This is an open access article under the CC BY license (http://creativecommons.org/licenses/by/4.0/).

Gould introduced a thought experiment that if the "tape of life" were replayed, evolutionary outcomes would differ profoundly (Gould, 1989). For a long period, parallel and convergent evolution were thus considered rare exceptions to evolutionary patterns rather than the norm.

In recent years, however, numerous examples of convergence and parallelism have been reported, prompting a reevaluation of their significance in evolutionary biology (Morris, 1998; 2003; Mcghee, 2011; Mahler et al., 2013; Stern, 2013; Bolnick et al., 2018; Zanne et al., 2018; Powell, 2020; Hjertaas et al., 2023; Ma et al., 2023). This has also intensified debates regarding the distinction between convergence and parallelism. Convergence refers to the evolution of similar traits in distantly related lineages, whereas parallelism occurs in closely related lineages. However, drawing a line between them is difficult (Arendt and Reznick, 2008; Rosenblum et al., 2014; Cerca, 2023; James et al., 2023). First, determining whether lineages are distantly or closely related can be challenging and often depends on the context. For example, *Arabidopsis thaliana* ecotypes Col-0 and C22 are closely related compared with *A. lyrata*, but *A. thaliana* and *A. lyrata* appear closely related when compared with *Populus*. Second, different genetic processes can produce similar traits, also blurring the line (Rosenblum et al., 2014; James et al., 2023). Moreover, evolution is rarely just one or the other, and convergence and parallelism may be better viewed as parts or endpoints of a quantitative continuum (Bolnick et al., 2018). This article is not intended to delineate these distinctions; for a deeper understanding, please refer to prior reviews (Arendt and Reznick, 2008; Stern, 2013; Rosenblum et al., 2014; Bolnick et al., 2018; Cerca, 2023; James et al., 2023). Instead, for simplicity, we adopt Arendt and Reznick's suggestion that all cases of independently derived similarity be referred to as convergent evolution, focusing on their functional and adaptive significance.

On the other hand, the increasing reports of convergence have revitalized the argument that evolution under similar circumstances may be more predictable than previously thought (Stern, 2013). Notable examples include convergent evolution of annual life in temperate regions, C_4 photosynthesis in arid or semiarid regions, and mangrove species in intertidal zones (Sage et al., 2011; Heyduk et al., 2019; He et al., 2022; Hjertaas et al., 2023). Our recent study also revealed genomic convergence across different plant lineages inhabiting terrestrial soils, characterized by gene tandem duplications (TDs) related to enzymatic catalysis and biotic stress responses, likely adaptations to soil microbial pressures (Wu et al., 2024a). To our knowledge, this represents the most widespread example of convergent evolution documented to date. This finding also aligns with established theoretical perspectives in evolutionary biology. As Simon Conway Morris noted, "The evolutionary routes are many, but the destinations are limited" (Morris, 2003), and similarly, George R. McGhee, Jr. proposed that convergent evolution arises from a finite set of solutions constrained by physics and geometry (Mcghee, 2011). These perspectives suggest that although evolution can take numerous paths, there are a limited number of optimal solutions to similar challenges, which implies that convergence is more the rule than the exception. Nevertheless, evolutionary outcomes are also influenced by historical contingency—unique events such as random mutations, genetic drift, or environmental disturbances that make outcomes idiosyncratic and non-repeatable (Gould, 1989; Blount et al., 2018). For an understanding of the interplay between contingency and convergence, please refer to the reviews Orgogozo (2015); Powell and Mariscal (2015); Blount et al. (2018) and Powell (2020).

Among the various environmental pressures driving convergent evolution, low temperatures stand out as a critical factor. The adaptation of angiosperms to frost zones provides a compelling case. Here, we focus on observations and implications of convergent adaptations in angiosperms (flowering plants) in cold climates. Angiosperms, which originated in warm, understory habitats (Feild et al., 2004; Soltis et al., 2008), have evolved into the most diverse and widespread clade, comprising nearly 90% of all extant land plant species (Crepet and Niklas, 2009). They dominate most ecosystems and biomes across nearly every climate, including the extremely cold regions of arctic and alpine tundra (Körner, 2021; Wang et al., 2023a).

This article reviews the replicated traits and molecular mechanisms by which angiosperms have adapted to cold environments. First, we investigate terrestrial regions exposed to chilling and freezing temperatures, angiosperm species in these areas, and their phylogenetic relationships. We next discuss key traits like herbaceousness and deciduousness that evolved independently in various plant lineages, likely as adaptations to frost zones. We also delve into the evolution of cold acclimation across species and its underlying pathways and discuss convergent evolution at multiple molecular levels, with a focus on the role of polyploidization in cold adaptation. Finally, we explore the artificial domestication of plants in frost zones and consider the potential impacts of global warming on plant migration and adaptation.

GLOBAL LANDSCAPE OF FROST REGIONS AND ASSOCIATED ANGIOSPERM FAMILIES

Global temperatures exhibit significant variation, impacting plant growth, survival, and distribution (Lancaster and Humphreys, 2020). Low temperatures can be categorized into chilling (0°C–15°C) and freezing (<0°C) (Schubert et al., 2020). Chilling temperatures primarily affect plants by reducing membrane fluidity, destabilizing photosynthetic components, and altering gene expression and protein synthesis, thus slowing growth and development or even causing lethal injury (Allen and Ort, 2001; Nievola et al., 2017). By contrast, freezing temperatures generally cause severe damage through ice crystal formation in plants (Sakai and Larcher, 1987; Pearce, 2001; Schubert et al., 2020). This process can lead to cellular dehydration, membrane rupture, and organelle destruction, ultimately resulting in structural damage, impaired functions, and potential cell death. Therefore, the threat posed by freezing often exceeds that of chilling stress and significantly restricts the geographic distribution of plant communities.

Geographic distribution and land coverage of low temperatures

As expected, the mean daily minimum air temperature of the coldest month (BIO6) (Karger et al., 2017) exhibits a

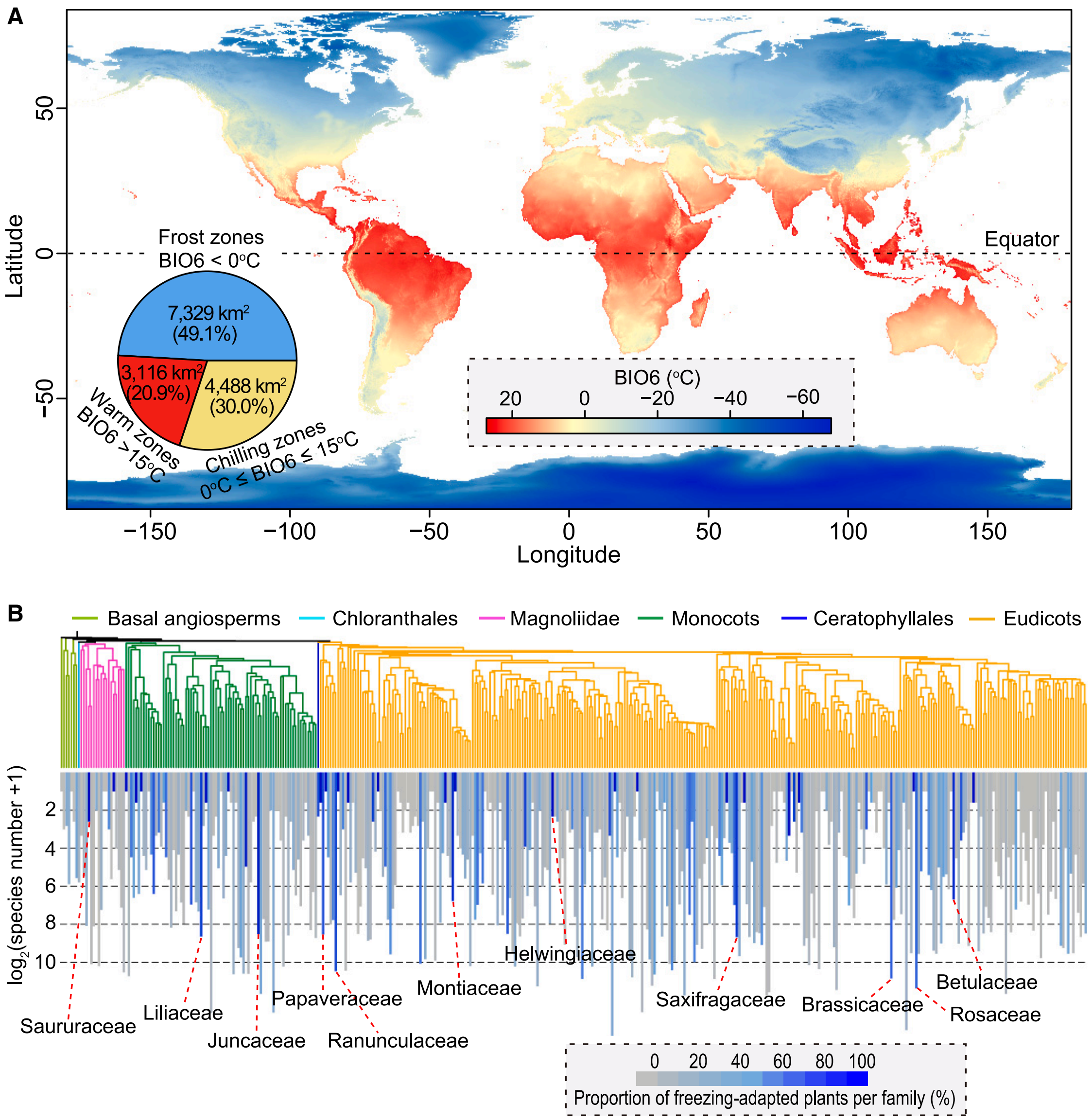

Figure 1. Global landscape of frost zones and associated angiosperm families.

(A) A global map of the mean daily minimum air temperature of the coldest month (BIO6). BIO6 data across the globe at 30 arc-second resolution (1979–2013) were sourced from the PaleoClim database (www.paleoclim.org, *Anthropocene*, v.1.2b**) (Karger et al., 2017) and used to categorize territorial lands into three distinct zones: frost zones with periodic or constant freezing (BIO6 < 0°C), chilling zones (0°C ≤ BIO6 ≤ 15°C), and warm zones (BIO6 > 15°C).

(B) Species number and proportion per angiosperm family in frost zones. Below the angiosperm family tree, a bar graph shows the log2-transformed species count for each family in frost zones. The bars are shaded to indicate the proportion of species in each family that can inhabit frost zones, with darker shades representing higher proportions. Geographic data for species were collected from GBIF (doi.org/10.15468/dL.4c2nfn), and each species had at least 10 valid records. Species, genus, and family names were validated using World Flora Online (WFO), yielding a dataset of 152 552 species, 11 391 genera, and nearly all angiosperm families (412 out of 416). A species was considered capable of inhabiting frost zones if it had at least five valid geographic records in these areas. The phylogenetic tree was constructed using the V.PhyloMaker2 R package (Jin and Qian, 2022) based on the APG IV system (Chase et al., 2016).

latitudinal gradient, with temperatures decreasing from the equator to the poles (Figure 1A). Our statistical analysis showed that nearly 80% of the Earth's terrestrial land surface is potentially affected by low temperatures, with 49.1% experiencing either periodic or constant freezing, broadly defined here as frost zones. The frost zones largely overlap with the Holarctic realm, home to diverse plant species adapted to cold and temperate climates (Liu et al., 2023). In such regions, freezing temperatures act as climatic filters, exerting strong selective pressure on life forms and shaping species persistence and adaptation strategies (Gutschick and Bassirirad, 2003; Donoghue and Edwards, 2014). Consequently, plant species in frost zones are often regarded as adapted to freezing zones.

Associated angiosperm families in frost zones

Temperate and polar zones are generally characterized by colder, drier, and more seasonally variable temperatures. Despite extreme cold and short growing seasons, many angiosperm lineages and species have independently adapted to these frost regions (Ricklefs and Renner, 1994; Preston and Sandve, 2013; Kerkhoff et al., 2014; Zanne et al., 2014; Spriggs et al., 2015; Cousins-Westerberg et al., 2023; Carruthers et al., 2024). Using species distribution data from GBIF (doi.org/10.15468/dL.4c2nfn), we compiled a dataset of 152 552 species with more than 201 million records, representing approximately half of all known angiosperm species and nearly all angiosperm families (412 out of 416) (Chase et al., 2016). Among these, 298 families are found in frost zones, and 137 families have over 90% of their species thriving in these areas (Figure 1B). For example, families such as Saururaceae, Liliaceae, Juncaceae, Papaveraceae, Ranunculaceae, Montiaceae, Helwingiaceae, Saxifragaceae, Brassicaceae, Rosaceae, and Betulaceae have an exceptionally high proportion of species (almost 100%) that can inhabit frost zones. This demonstrates the various degrees of specialization and evolutionary adaptation to cold climates among angiosperm families, a significant number of which convergently exhibit strong adaptations to frost conditions.

Like high-latitude regions, high-altitude areas such as the Qinghai–Tibetan Plateau and the Andes, Alps, and Rocky Mountains are often exposed to freezing temperatures (Barry, 2008). Many studies have investigated plant communities and their adaptive traits in these environments (Körner et al., 1989; Gale, 2004; Halbritter et al., 2018; Liu et al., 2020). Using data from a study of the Qinghai–Tibetan Plateau (Zhang et al., 2016), we identified 2863 angiosperm species that occur above the timberline (Figure 2A). These species span a wide range of monocots and eudicots, with significant representation from angiosperm families such as Poaceae, Ranunculaceae, Asteraceae, and Rosaceae (Figure 2B). Examples of alpine species include *Deschampsia cespitosa* (Poaceae), *Thalictrum alpinum* (Ranunculaceae), *Aster alpinus* (Asteraceae), and *Malus transitoria* (Rosaceae). The distant evolutionary relationships of these species highlight their independent adaptive evolution to thrive in extreme alpine environments. Notably, high-altitude regions exhibit a significantly higher proportion of herbaceous plants than woody plants (Figure 2A). The harsh conditions in frost zones have exerted strong selective pressures on plant traits such as herbaceousness and deciduousness. In the following section, we will examine these traits in relation to their potential adaptations to freezing temperatures.

ADAPTIVE STRATEGIES FOR FREEZING TEMPERATURES

In the face of freezing temperatures, plants have developed a range of adaptive strategies, including evasion, avoidance, and tolerance (Levitt, 1980; Sakai and Larcher, 1987; Vitasse et al., 2014). Freezing evasion involves completing the life cycle or entering dormancy before the onset of frost, thereby protecting sensitive tissues from damage. Herbaceous plants, for example, often overwinter as dormant seeds or through underground storage tissues (e.g., rhizomes, bulbs, or tubers), which are shielded from extreme temperatures by the insulating effects of snow and soil (Preston and Sandve, 2013; Vitasse et al., 2014). Woody plants, on the other hand, employ a more complex set of mechanisms. Deciduous trees, for example, use evasion by shedding their leaves in autumn to reduce the risk of frost damage. However, their aboveground structures must rely on additional strategies, such as freezing avoidance or tolerance, to survive low temperatures. Freezing avoidance prevents ice formation in tissues by supercooling fluids, keeping water in a liquid phase at sub-zero temperatures (Levitt, 1980; Vitasse et al., 2014). By contrast, freezing tolerance allows ice crystals to form without causing lethal damage to cell structures by limiting crystal size, controlling the location of ice formation, and mitigating the dehydration caused by extracellular freezing (Vitasse et al., 2014; Bredow and Walker, 2017; Schubert et al., 2020). Our understanding of freezing avoidance and tolerance, collectively referred to as freezing resistance, is based largely on studies of model plants, which often enhance their freezing resistance through cold acclimation (Thomashow, 1999; Kidokoro et al., 2022)—a process that will be discussed in the next section. This section focuses on evasion adaptations, especially the herbaceous form and leaf deciduousness.

Herbaceous form

Angiosperms can be broadly classified into woody and herbaceous plants, although this distinction is somewhat artificial (Groover, 2005). When considering the presence of cambium and secondary growth, woody plants are polyphyletic, meaning they are dispersed across multiple lineages rather than confined to a single monophyletic group. From an evolutionary perspective, the ancestral states of angiosperms are thought to have been woody species that originated in warm, understory habitats (Feild et al., 2004; Soltis et al., 2008). Over time, multiple woody lineages independently experienced loss or partial reduction of the vascular cambium and secondary growth, giving rise to herbaceous plants (Groover, 2005; Klimeš et al., 2022). By contrast, many herbaceous lineages have also re-evolved into woody forms (Nürk et al., 2019; Zizka et al., 2022). These frequent evolutionary transitions suggest the adaptive advantages of both growth forms in their respective niches.

Our analysis revealed a significant positive correlation between frequency of herbaceous species and distance from the equator, which is strongly associated with the coldest month temperature (BIO6) ($p = 1.8 \times 10^{-8}$, pseduo-R^2 = 58.6%) (Figure 3A). This trend is supported by previous studies (Moles et al., 2009; Massante et al., 2019). Further eco-environmental analysis incorporating climatic, edaphic, topographic, and anthropogenic variables (n = 62) identified temperature-related variables (e.g., BIO11, BIO3, BIO6, and BIO1) as the primary factors associated with the distribution of plant growth habits (Supplemental Figure 1A). Notably, many angiosperm lineages, especially those in monocots, Caryophyllales, and Lamiales, have convergently evolved herbaceous habits, regardless of their distinct phylogenetic affiliations (Figure 3B). This convergent evolution is exemplified by herbaceous species such as *A. lyrata*, *Astragalus alpinus*, *Stellaria crassifolia*, *Papaver radicatum,* and *Corallorhiza trifida*, which belong to

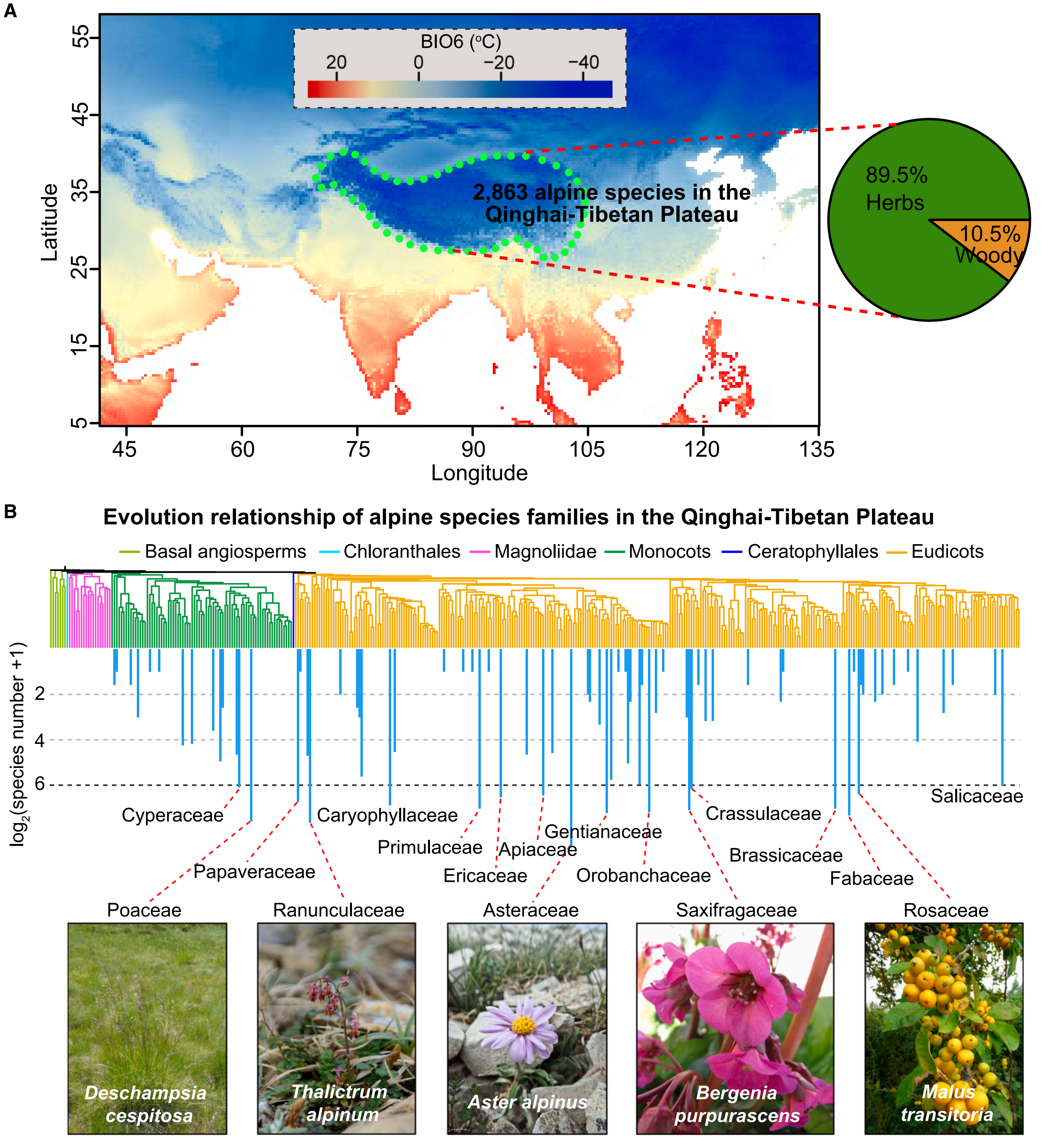

Figure 2. Independent adaptation of angiosperms in the Qinghai–Tibetan Plateau.

(A) Numbers and growth forms of alpine species found above the timberline in the Qinghai–Tibetan Plateau. These species were sourced from the study of Zhang et al. (2016), and only the angiosperm species validated by WFO were retained, totaling 2863. Their growth forms (herbs or woody) were identified using WorldFloraDB (www.worldfloradb.net).

(B) Independent adaptation of alpine species in angiosperm families. Below the angiosperm family tree, a blue bar graph shows the log2-transformed species count for each family observed in the Plateau. Species photographs, such as those of *Deschampsia cespitosa*, *Thalictrum alpinum*, *Aster alpinus*, *Bergenia purpurascens*, and *Malus transitoria*, are courtesy of the following sources: European Environment Agency (https://eunis.eea.europa.eu/species/190299), Mike Pennington (geograph.org.uk/photo/2413946), Amphithoe (flickr.com/photos/amphithoe/27875401342), HQ Flower Guide (flickr.com/photos/nhq9801), and Jonathan Billinger (geograph.org.uk/photo/978786), respectively.

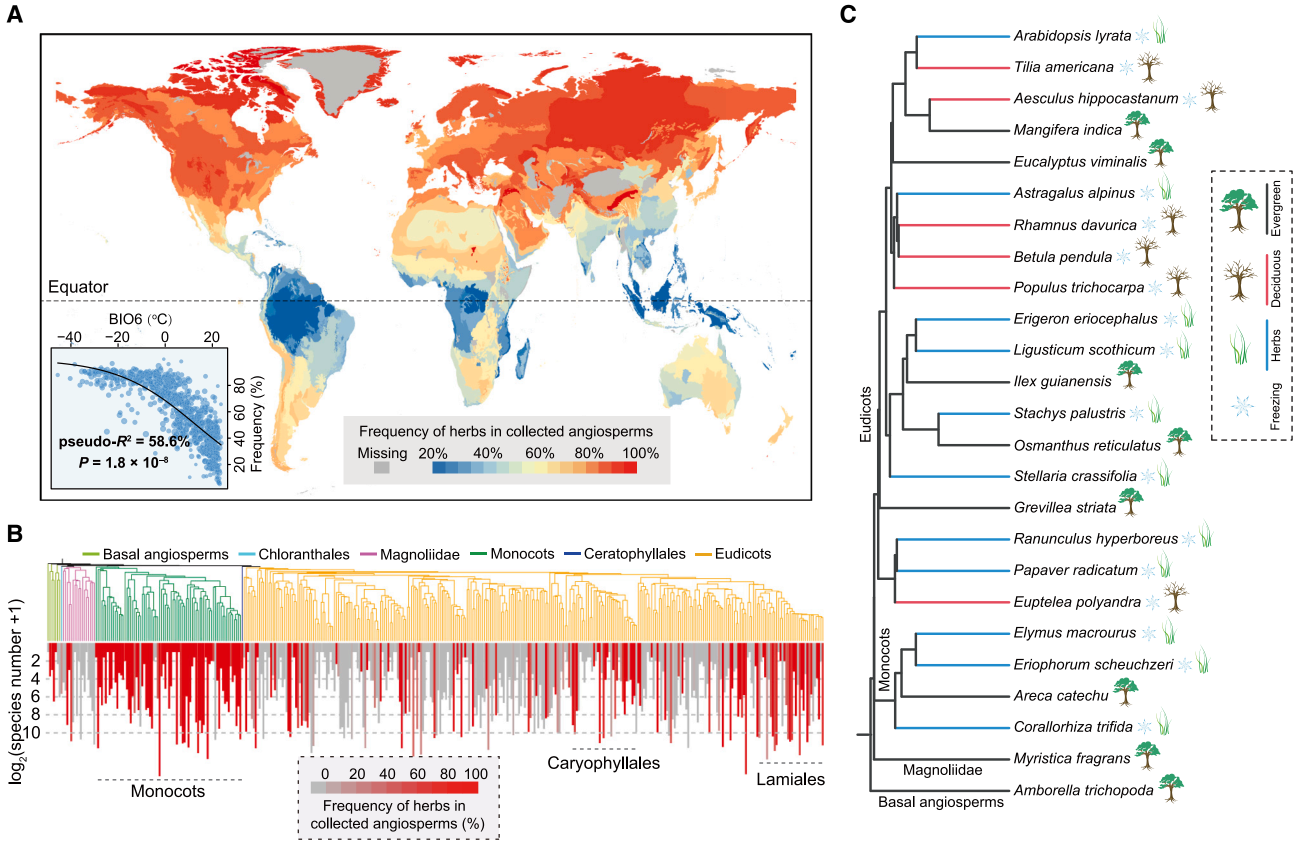

Figure 3. Convergent evolution of herbaceous species in freezing temperatures.

(A) An ecoregion-level map of herbaceous plant frequency. The terrestrial landscape was subdivided into 827 ecoregions based on the World Wildlife Fund (WWF) classification (Olson et al., 2001). After geographic data cleaning and control (Rice et al., 2019) and annotation of growth forms using public data (Zanne et al., 2014; Weigelt et al., 2020), a total of 67 961 herbaceous and 55 557 woody angiosperm species were identified. Herbaceous plant frequency was calculated as the proportion of herbaceous species relative to the total number of herbaceous and woody angiosperm species within each ecoregion. In the bottom left corner, the relationship between herbaceous frequency and BIO6 is evaluated by univariate beta regression; its significance was assessed using a modified *t*-test that accounted for spatial autocorrelation (Clifford et al., 1989). In addition, an ecoregion-level map of deciduous plant frequency is shown in Supplemental Figure 2.

(B) Independent evolution of herbaceous growth form in angiosperms. Angiosperm families were used to construct this phylogenetic tree. Below the angiosperm family tree, a bar graph shows the log2-transformed number of herbaceous species per family in frost zones. The bars are shaded to indicate the proportion of herbaceous species within each family that can inhabit frost zones, with darker shades representing higher proportions. A species was considered capable of inhabiting frost zones if it had at least five valid geographic records in these areas, and only ecoregions with 20 or more species were included in the analysis.

(C) Evolutionary relationships between herbaceous and deciduous woody species found in frost zones and their tropical/subtropical evergreen relatives.

different angiosperm lineages but are primarily distributed in temperate, subarctic, and/or arctic regions characterized by cold environments (Figure 3C). Similarly, the presence of herbaceous species from diverse families in high-altitude regions with cold climates, such as the Qinghai–Tibetan Plateau, further supports this perspective (Figure 2B). Herbaceous species, whether annual or perennial, can cope with frost damage by overwintering as dormant seeds or underground organs. A recent study found that herbaceous plants can occupy regions with lower temperatures, although they experience a shorter growing season, with an average of 132.7 frost-free days compared with 205.3 days for woody plants (Klimeš et al., 2022). Thus, the convergent evolution of the herbaceous form may contribute to plant adaptation in colder environments.

The question of whether the occupation of cold climates preceded the evolution of the herbaceous form, or vice versa, is intriguing. Through phylogenetic reconstruction, Zanne et al. (2014) predicted that 58% of herbaceous species had already transitioned to the herbaceous form before encountering freezing temperatures. Although the method used in that study has been contested (Edwards et al., 2015), both phenomena—transitions into freezing temperatures before or after the evolution of the herbaceous form—may be frequent, similar to the repeated evolutionary transitions in growth form between herbaceous and woody species (Nürk et al., 2019; Klimeš et al., 2022; Zizka et al., 2022). Herbaceous species generally have higher adaptability to low temperatures than woody species, resulting in the distribution pattern of herbaceous plants that we observe today. Moreover, herbaceous genera have diversified at a much faster rate than woody genera during the past 30 million years of the Late Cenozoic Ice Age compared with their diversification during global warming periods (Lu et al., 2018). These data provide evidence that the herbaceous

form is an adaptive trait in cold environments, regardless of whether its evolution occurred before or after transitions into cold environments.

Many warm regions, such as tropical rainforests, also host a variety of herbaceous species, especially in the understory or margins of forests where light can penetrate (Whitmore, 1990). This suggests that the herbaceous form is also beneficial in other environments beyond those with freezing temperatures. Herbs generally exhibit a higher growth rate and possess less costly aboveground structures, with some being shade tolerant (Augspurger and Salk, 2017; Klimeš et al., 2022). This allows herbaceous species to adapt effectively in environments where they cannot directly compete with woody species, such as trees, for sunlight. In addition, the rapid growth of herbs, such as *Spartina alterniflora* and *Phragmites australis*, enables them to thrive in new environments (Bowen et al., 2017; Hao et al., 2024). This adaptability highlights the strategic advantages of herbaceous plants, such as rapid growth and/or some shade tolerance, which enable them to thrive in diverse ecological settings beyond freezing temperatures.

Leaf deciduousness

Unlike herbaceous species, woody species, arbitrarily considered those with persistent aboveground stems, appear to adopt a more complex strategy in response to freezing exposure (Zanne et al., 2014; Wisniewski et al., 2018). For instance, deciduous woody species shed their leaves while retaining stems, combining evasion and resistance strategies to survive freezing winters. This process not only reduces water loss and energy expenditure for dormancy during the cold season but also ensures resilience and supports rapid growth in the following year (Vitasse et al., 2014).

Deciduous leaf phenology, which dominates northern temperate forests, has independently evolved numerous times across different plant lineages, representing one of the most striking examples of convergent evolution (Edwards et al., 2017; Folk et al., 2020). Similar to the distribution of herbaceous species frequency (Figure 3A), the presence of deciduous woody species follows a latitudinal gradient and shows a strong association with the coldest month temperatures (BIO6) (p = 1.9 × 10^{-7}, pseduo-R^2 = 49.0%) (Supplemental Figure 2A). Eco-environmental analysis further identified temperature-related variables (e.g., BIO4, BIO6, BIO11, and BIO7) as the primary factors influencing the distribution of deciduous leaf phenology (Supplemental Figure 1B). As expected, temperature seasonality (BIO4) and temperature annual range (BIO7) were positively associated with the prevalence of deciduous woody species. Notably, many deciduous woody species from diverse lineages—particularly those in Magnoliaceae, Grossulariaceae, Cornaceae, Caprifoliaceae, Rosaceae, Betulaceae, and Salicaceae—have independently evolved deciduousness regardless of their family affiliations (Supplemental Figure 2B). For instance, species like *Aesculus hippocastanum*, *Populus trichocarpa*, and *Euptelea polyandra* evolved in different lineages but are predominantly distributed across temperate and subarctic regions characterized by distinct seasons and periodic freezing temperatures (Figure 3C). Thus, freezing temperatures likely represent a key selective pressure for the convergent evolution of deciduous trees and shrubs.

Deciduousness is also regarded as an adaptation in other stress-related environments, such as dry tropical forests and seasonally dry Mediterranean regions, where drought, rather than cold, determines the unfavorable growth season (Murphy and Lugo, 1986; Folk et al., 2020). Fossil evidence indicates that angiosperm deciduous strategies originated in the Late Cretaceous and prevailed in high northern latitudes, during a time when polar regions experienced subtropical conditions characterized by drought and prolonged darkness rather than freezing (Axelrod, 1966; Wolfe and Upchurch, 1986). It is thus explained that in environments where water or sunlight was limited, deciduousness helped plants survive by shutting down hydraulic function, reducing water loss, and conserving resources. As cold climates became widespread, this pre-existing trait also proved advantageous, allowing plants to adapt more readily to the new, cold conditions. Therefore, it is likely that the ability to shed leaves initially evolved as an adaptation to drought or other stresses and was later co-opted as a beneficial trait in cold climates (Wolfe and Upchurch, 1986; Edwards et al., 2017).

However, Zanne et al. (2014) predicted that most angiosperm lineages initially established themselves as evergreen plants in freezing environments, later evolving deciduousness. They also revealed that the distribution of deciduous trees and shrubs is significantly correlated with temperature but not significantly associated with precipitation (Zanne et al., 2018). This temperature-associated pattern was further supported by another study (Ma et al., 2023) and our own analysis, although our results indicate that precipitation has a lesser, yet still significant, impact on the distribution of deciduous trees (Supplemental Figure 1B). Although the sequence of deciduousness and climate transitions is debated, deciduous leaf phenology is considered an adaptive trait that confers a survival advantage to angiosperm woody species in environments with periodic freezing conditions (Zanne et al., 2014, 2018; Edwards et al., 2017; Ma et al., 2023).

Other traits

In addition to leaf phenology, small hydraulic conduits (xylem vessels and tracheids) may reduce the likelihood of ice crystal formation in woody plants. There is a trade-off between vascular efficiency and the risk of freeze–thaw embolism (Davis et al., 1999), with smaller conduits playing a critical role in reducing this risk. For many deciduous woody species from diverse families in cold climates, hydraulic conduit size likely decreases with declining minimum temperatures, while the presence of deciduous leaves increases (Zanne et al., 2014, 2018; Ma et al., 2023).

Furthermore, plants in regions with periodic freezing have developed other adaptive traits. In alpine environments, different species such as *Myosotis alpestris*, *Geum rossii*, and *Saussurea involucrata* have evolved to flower rapidly during brief warm periods, allowing them to fully capitalize on the short growing season (Chik et al., 2015; Körner, 2021). Cold environments, particularly the Arctic tundra, also limit the activity of biotic pollinators (Kevan, 1972), and many flowering plants have

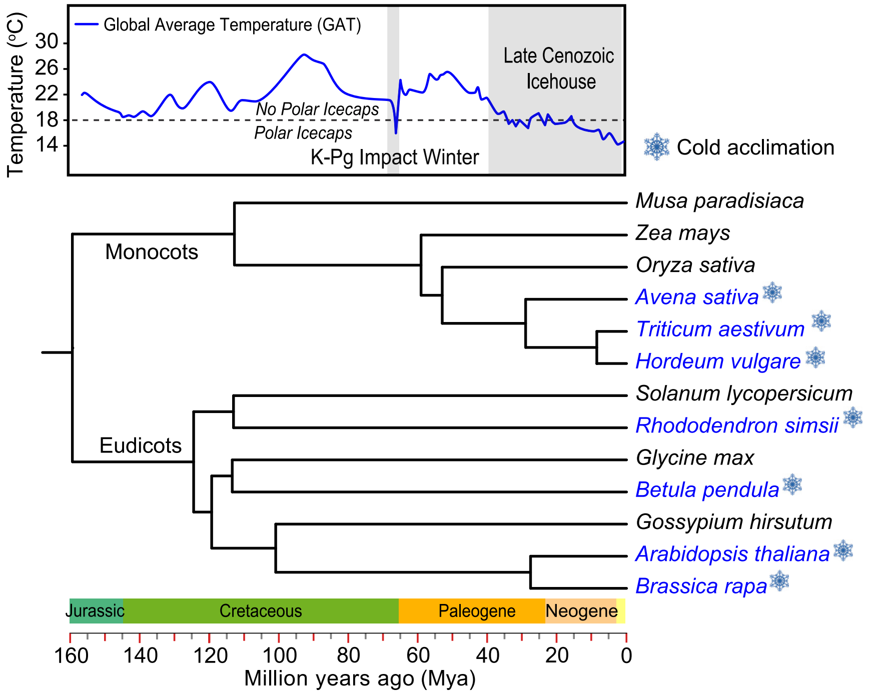

Figure 4. Convergent evolution of cold acclimation in selected angiosperm species.
The top curve illustrates fluctuations in global average temperature (GAT) over the last 160 million years (Scotese et al., 2021), marked by a sharp decline around the Cretaceous–Paleogene (K–Pg) boundary (~66 mya) and a continued decrease during the Late Cenozoic Ice Age. Large polar icecaps formed when the GAT dropped below 18°C (dashed line). Representative cold-acclimated and non-cold-acclimated species were obtained from previous studies (Chinnusamy et al., 2007; Schubert et al., 2019; Liu et al., 2022).

evolved to rely on wind pollination or self-pollination mechanisms to ensure reproductive success (Barrett, 2002; Friedman and Barrett, 2009; Wright et al., 2013; Rodger et al., 2021). Although herbaceous and deciduous woody species dominate in temperate regions, some evergreen plants, such as *Pinus pinaster* and *Quercus ilex*, have developed adaptations such as increased leaf thickness and cell wall rigidity to enhance freezing tolerance (González-Zurdo et al., 2016). Collectively, these examples highlight the influence of freezing temperatures in driving the evolution of similar adaptive traits or functions across diverse plant lineages.

EVOLUTION OF COLD ACCLIMATION

In addition to visible macroscopic traits, many plants in temperate, subarctic, and arctic regions have developed sophisticated molecular mechanisms to cope with cold stress (Kidokoro et al., 2022; Kerbler and Wigge, 2023). To mitigate cold damage, they produce cryoprotective proteins, such as dehydrins and antifreeze proteins, to prevent ice crystal damage and adjust membrane lipid composition for stability in freezing conditions (Kosová et al., 2019). They also synthesize antioxidant enzymes like catalase and superoxide dismutase to neutralize reactive oxygen species (Dreyer and Dietz, 2018). In addition, osmoprotectants, including proline and soluble sugars, contribute to osmotic adjustment (Saxena et al., 2013). These responses are orchestrated by complex stress response networks (Barrero-Gil and Salinas, 2017; Shi et al., 2018). A key process within these networks is cold hardening, or cold acclimation, which enables plants to sense gradually decreasing, nonfreezing temperatures and activate regulatory pathways, such as the C-repeat binding factor/dehydration-responsive element binding protein 1 (CBF/DREB1) pathway, to enhance freezing resistance (Pearce, 2001; Schubert et al., 2020). For a comprehensive overview of these processes, we refer readers to recent reviews (Ding et al., 2020; 2024; Zhang et al., 2022a; Kidokoro et al., 2022; Kerbler and Wigge, 2023). Our focus here is on the evolution of cold acclimation and its underlying complex mechanisms.

Independent evolution of cold acclimation

In contrast to temperate plants, tropical and subtropical plants such as rice (*Oryza sativa*), maize (*Zea mays*), and tomato (*Solanum lycopersicum*) are generally chilling sensitive and incapable of cold acclimation (Chinnusamy et al., 2007; Shi et al., 2018; Liu et al., 2022). This raises an intriguing question: have tropical species lost cold acclimation, or have temperate plants evolved it? Fossil and molecular data suggest that angiosperms originated in warm habitats and radiated since the Lower Cretaceous, thriving in high latitudes with subtropical conditions (Axelrod, 1966; Wolfe and Upchurch, 1986; Smith and Donoghue, 2008). This aligns with the warm climates during that era, in which global average temperatures exceeded >18°C (Figure 4). These results suggest that angiosperms originated and diversified in a warm environment, potentially unrelated to the evolution of cold acclimation.

Studies suggest that cold adaptations in temperate grasses and polar plants evolved independently after their divergence (Schubert et al., 2019; Birkeland et al., 2020, 2022). Our phylogenetic analysis of temperate and tropical/subtropical plants revealed that temperate plants likely developed cold acclimation capabilities independently in response to decreasing paleoclimatic temperatures (Figure 4). For instance, *Avena sativa*, *Triticum aestivum*, and *Hordeum vulgare* (Poaceae); *Rhododendron simsii* (Ericaceae); *Betula pendula* (Betulaceae); and *A. thaliana* and *Brassica rapa* (Brassicaceae) independently adapted to cold climates compared with their tropical/subtropical relatives during the Cretaceous–Paleocene (K–Pg) impact winter and Late Cenozoic Ice Age. Given the origin and radiation of the major angiosperm lineages in warm paleoclimates (Axelrod, 1966; Wolfe and Upchurch, 1986; Smith and Donoghue, 2008), cold acclimation likely evolved independently multiple times across different lineages after their divergence.

Complex mechanisms underpinning cold acclimation

The complex mechanisms underlying cold acclimation in plants involve multiple pathways, in particular the CBF/DREB1

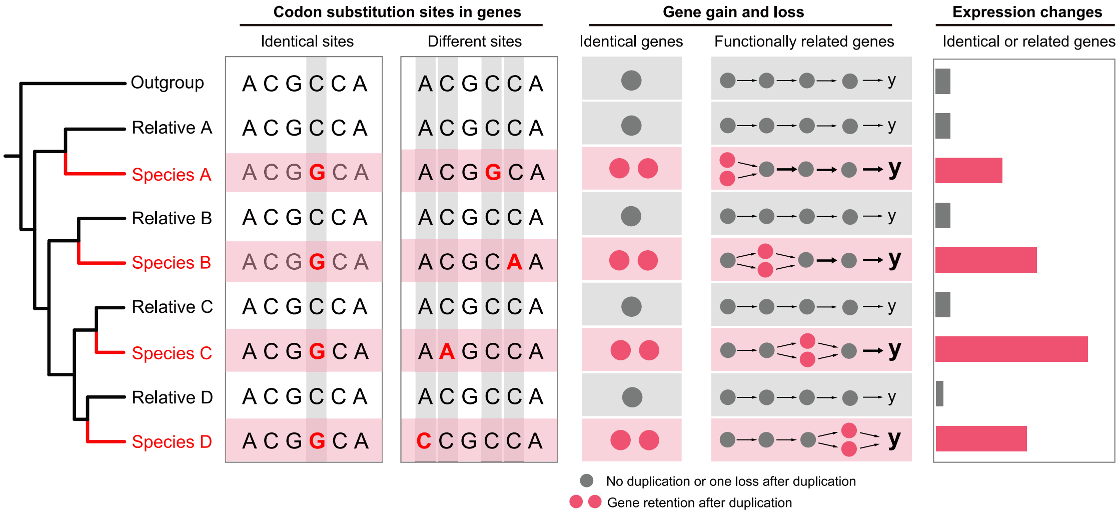

Figure 5. Genetic convergence for plant cold adaptation at multiple levels.
Across independent clades (species A–D), convergent evolution can occur at identical and different codon sites within genes, at identical genes, or at functionally related genes through gene gain or loss, as well as through changes in gene expression.

regulatory pathway (Ding et al., 2020; 2024; Zhang et al., 2022a; Kidokoro et al., 2022). Research has primarily assumed that CBF/DREB1 genes perform similar functions across species. However, despite their functional conservation, evolution has continuously progressed and continues to do so. The origins of CBF/DREB1 genes can be traced back to a TD of a DREB III gene, followed by an ε whole-genome duplication (WGD) that produced two archetypes, clades I and II, in ancient angiosperms (Nie et al., 2022). Clade II evolved to acquire cold-sensitive induction and expanded independently in eudicots and monocots during global cooling periods (Wu et al., 2020a; Guo et al., 2022; Nie et al., 2022). Subsequent post-duplication and evolutionary trajectories led to their functional divergence. For example, although certain CBF/DREB1 genes in rice and maize are strongly induced under chilling temperatures (Nie et al., 2022), their role in cold acclimation is moderate. Furthermore, unlike the preference of *Arabidopsis* AtCBF2/3/1 for the 5’-A/GCCGAC-3’ sequence, rice OsDREB1C binds specifically to 5’-GCCGAC-3’ (Song et al., 2021; Wei et al., 2022; Deng et al., 2024). This divergence, along with gene gains/losses and expression reprogramming in rice, contributes to the functional innovation of OsDREB1C in increasing nitrogen use efficiency, photosynthesis, and grain yield (Wei et al., 2022; Deng et al., 2024). These results suggest that although the CBF/DREB1 pathway is largely conserved, we cannot universally extrapolate their functions to all angiosperm species.

Furthermore, cold acclimation involves multiple pathways that extend beyond the CBF/DREB1 pathway. Notably, only 10%–20% of *cold-regulated* (*COR*) genes in cold acclimation are directly regulated by CBF/DREB1 (Park et al., 2015; Jia et al., 2016; Zhao et al., 2016; Song et al., 2021). Other transcription factors, such as HSFC1, ZAT12, ZF, ZAT10, CZF1, and BBX29, have been identified as parallel regulators of *COR* genes (Park et al., 2015; Wang et al., 2023b). The genetic and physical interactions among these transcription factors can lead to synergistic or antagonistic effects on gene expression, shaping the overall cold acclimation phenotype. Phytohormones such as abscisic acid and jasmonates also play roles in modulating cold responses, either independently or in coordination with transcription factors such as CBF/DREB1 proteins (Barrero-Gil and Salinas, 2017; Shi et al., 2018). Plants also integrate other signaling pathways, such as light signaling, vernalization, and the circadian clock, to fine-tune the regulation of *COR* gene expression (Kidokoro et al., 2022; Wang et al., 2023b; Guo et al., 2023; Ding et al., 2024). This intricate network of transcriptional regulators, hormonal signaling, and environmental cues suggests the adaptability and resilience of plants in cold environments, highlighting the complexity of cold acclimation processes across different species.

GENETIC CONVERGENCE FOR PLANT ADAPTATIONS

In this section, we discuss the potential for genetic convergence among plant genomes in adaptation to cold climates. Genetic convergence can occur at multiple molecular levels, such as substitutions at identical or different amino acid sites within genes, changes in gene duplication and expression, and the involvement of distinct genes within the same or similar functional pathways (Figure 5), with each potentially varying in its effect on convergent evolution.

Positive selection and convergent site substitution

Plant adaptation can occur through positive selection of advantageous mutations (Desalle, 2000; Vitti et al., 2013). However, compared with neutral and deleterious mutations, advantageous mutations are rare and can be lost through genetic drift (Eyre-Walker and Keightley, 2007), making them uncommon across genomes. In addition, many detected instances may be false positives due to statistical errors (Storey and Tibshirani, 2003). Convergent site substitution, in which identical amino acid changes occur at the same site under positive selection across species or populations, is a powerful example of natural selection (Wu et al., 2020b; Xu et al., 2020).

Nevertheless, the number of positively selected sites is inherently limited, and cases in which identical amino acid changes occur and become fixed independently across species are even rarer. For example, a study on the genetic convergence of marine mammal genomes, including those of manatees, walruses, and killer whales, found that among 661–732 genes under positive selection, only eight exhibited identical amino acid substitutions across all three lineages (Foote et al., 2015).

Compared with animals, plants generally experience more frequent polyploidization events and have larger multigene families, resulting in complex networks of orthologs and paralogs between and within species (Murat et al., 2012; Wang et al., 2023b). This complexity poses challenges for identifying orthologous genes across species and analyzing their potential for positive selection and convergent site substitution. Despite these challenges, research has been performed in this area. A notable example is the independent evolution of C_4 photosynthesis in plants, in which multiple sites in C_4-related genes such as *PEPC* and *rbcL h*ave undergone positive selection and converged to similar or identical amino acid changes across different C_4 grass lineages (Christin et al., 2007, 2008). To address the noise from rapidly evolving sites and falsely inferred convergence, Xu et al. (2017) developed a method called convergence at conservative sites. Using this method, they identified approximately 400 genes containing candidate adaptive convergent sites in three mangrove species (Xu et al., 2017). Further refinement of the method enabled them to identify 73 convergent genes potentially involved in mangrove salinity tolerance in intertidal zones (He et al., 2020). However, most of the identified convergent site substitutions were limited to two of the three mangrove species, with very few shared across all three.

Studies on convergent adaptation to cold climates have also found limited evidence of substitutions at identical sites within genes. For example, Birkeland et al. (2020) found little evidence of convergent substitutions when examining three Brassicaceae species (*Cardamine bellidifolia*, *Cochlearia groenlandica*, and *Draba nivalis*) that colonized the Arctic. By contrast, without requiring substitutions to occur at identical sites, Zhang et al. (2023) identified 36 genes under convergent positive selection in three to five out of seven distantly related alpine plant species. Similarly, an analysis of 9 million single-nucleotide polymorphisms (SNPs) across 18 natural populations of three Brassicaceae species (*Arabis alpina*, *Arabidopsis halleri*, and *Cardamine resedifolia*) revealed 298 genes with non-synonymous SNPs potentially involved in convergent adaptation to alpine environments, although most sites of SNPs differed in genes among species (Rellstab et al., 2020). These results highlight the rarity of identical amino acid changes in abiotic stress responses, including cold adaptations. Instead, convergent evolution more commonly involves changes at different amino acid sites within genes across species.

Gene duplication through polyploidization and tandem duplication

Although site-specific convergence appears to be limited, a substantial number of duplicated genes, as well as many distinct duplicated genes functioning in similar stress response pathways, have been identified as potential convergent adaptations to extreme abiotic stresses, such as cold climates (Birkeland et al., 2020; Zhang et al., 2023). To our knowledge, angiosperm genomes are generally abundant in gene duplicates, primarily owing to historical WGDs, also known as polyploidization events, that have occurred recurrently over the past 200 million years of angiosperm evolution (Wu et al., 2020a; Zhang et al., 2020; Van de Peer et al., 2021; Carruthers et al., 2024). WGD results in a doubling of genome content, providing vast genetic resources for gene convergent evolution across different plant lineages during adaptation to similar environmental pressures.

In natural environments, polyploid frequency increases with latitude, especially in frost zones, such as the tundra biome of the far northern hemisphere (Brochmann et al., 2004; Martin and Husband, 2009). A comprehensive analysis of tens of thousands of angiosperm species confirmed a clear latitudinal trend in global polyploidy distribution (Rice et al., 2019). In addition, polyploids are prevalent in high-altitude regions, where environmental conditions often overlap with those of cold climates (Schinkel et al., 2016). This suggests that polyploidy plays a role in plant adaptation to frost zones. For instance, species such as *Hedyotis caerulea*, *Lolium perenne*, *Nicotiana benthamiana*, and *Plumbago articulata* have demonstrated cold tolerance as polyploids (Sugiyama, 1998; Deng et al., 2012; Jiang et al., 2020). Notably, colchicine-induced octaploids of *N. benthamiana* exhibited a 70% increase in survival under cold stress, and a synthetic tetraploid of *P. auriculata* demonstrated greater cold tolerance than its diploid counterparts (Deng et al., 2012; Jiang et al., 2020).

Interestingly, alongside polyploid plants, many diploid plants found in high latitudes and altitudes may also have experienced historical WGD. Because long-term retention of WGD is rare, likely owing to minority cytotype exclusion (Comai, 2005), most genes revert to a single-copy state over time through fractionation and diploidization processes (Lynch and Conery, 2000). By contrast, retained gene duplicates are considered particularly important for genetic innovation and adaptation to environmental challenges (Force et al., 1999; Song et al., 2020). For example, a wave of WGD events occurred independently across many angiosperm lineages around the K–Pg boundary, coinciding with global cooling (Schulte et al., 2010; Vanneste et al., 2014; Guo et al., 2024). Another wave of more recent WGD events took place during the global cooling in the Late Cenozoic Ice Age (Wu et al., 2020a; Nie et al., 2022). Following these events and throughout the diploidization process, many duplicated genes associated with stress responses, especially those related to cold stress, were convergently retained across different lineages (Wu et al., 2020a; Nie et al., 2022; Guo et al., 2024). Key retained genes included cold-responsive regulatory factors such as *CBF2/4*, *ICE1/2*, *GRP7/8*, *CCA1/LHY*, and *RVE4/8*. Their downstream regulons, like *GOLS2/3*, *CAX1/3*, and *LEA2/5*, were also convergently retained. In ancient angiosperms, the cold-responsive regulatory network was simple, but through the processes of polyploidization, diploidization, and natural selection, it was significantly rewired and strengthened (Wu et al., 2020a; Song et al., 2020; Guo et al., 2022, 2024; Nie et al., 2022). Overall, WGD is more like a temporary transitional state that provides gene duplicates for the rewiring of gene regulatory networks under natural selection. This

process contributes to the complexity and robustness of stress-related networks for plant adaptation in cold climates.

Despite the occurrence of historical WGD in many angiosperm lineages, a substantial number of eudicot lineages, such as *Rosa*, *Betula*, and *Vitis*, did not experience WGD during the cooling periods of the K–Pg boundary and the Late Cenozoic Ice Age (Guo et al., 2022). Notably, some lineages not only survived the severe paleoenvironmental cooling but also demonstrated a strong ability to adapt to extreme cold stress. For example, *B. pendula* thrives in temperate and subarctic regions, exhibiting significant cold tolerance (Salojärvi et al., 2017; Cheng et al., 2024). Research suggests that small-scale duplications, particularly TDs, may provide an alternative mechanism to WGD for the replication of stress-related family genes, especially AP2/ERF III and IX genes such as *CBF/DREB1* (Salojärvi et al., 2017; Guo et al., 2022). During similar periods of global cooling, many other cold-responsive genes, such as *RD29 A/B*, *COR15 A/B*, and *KIN1/2*, were also expanded by TD (Guo et al., 2024). This offers a significant mechanism for gene duplication and genetic innovation in plants, especially for those species that have not undergone WGD during global cooling periods.

Changes in gene expression

Compared with variations in amino acid sites and gene duplicates, gene expression regulation has an immediate and direct effect on phenotypes (Xu et al., 2020). Under low temperatures, many genes are upregulated to enhance cold tolerance by producing enzymes and proteins for osmolyte synthesis, metabolism, and transport, whereas genes related to photosynthesis, such as those encoding photosystem I and II subunits, are downregulated (Chinnusamy et al., 2007; Maruyama et al., 2012; Guo et al., 2023). Thus, the evolution of gene expression in different plants may convergently contribute to cold tolerance. For example, distantly related species, such as carrot, *D. antarctica*, and *L. perenne*, exhibit convergent evolution in the expression of antifreeze proteins upon exposure to low temperatures, albeit with different levels of accumulation (Gupta and Deswal, 2014). Epigenetic mechanisms, such as DNA methylation and histone modifications, also play a significant role in cold responses. For instance, in upland cotton (*Gossypium hirsutum*), cold treatment results in a global decrease in DNA methylation, leading to increased expression of defense-related genes (Fan et al., 2013). Similar changes in DNA methylation occur in the rubber tree (*Hevea brasiliensis*) under cold stress (Tang et al., 2018).

These studies suggest that plant adaptation to cold environments encompasses a diverse range of convergent evolutionary changes. These range from relatively rare identical genetic changes to extensive convergence in genes, stress response pathways, and gene expression. In addition, some convergence is observed at different sites within genes under positive selection across different species. However, variation in the contribution of these different types of change to convergent evolution is not absolute and may be influenced by multiple factors, including the analytical methods used, the phylogenetic distance between species, and the different focuses of the studies. More detailed comparative studies are required to better understand the relative importance of these different modes of molecular convergence.

ARTIFICIAL DOMESTICATION OF PLANTS IN COLD CLIMATES

Many species, including crops, medicinal plants, and horticultural plants, have been independently adapted by humans to grow in cold climates, despite their origins in warm regions (Doebley et al., 2006; Chen et al., 2022). For example, cold adaptation of *japonica* rice has been facilitated by the selection of the *COLD1* gene (Ma et al., 2015). For maize, the selection of genes like *ZmPRR7* promotes early flowering, facilitating adaptation to higher latitudes and altitudes (Chen et al., 2022). Similarly, many traditional Chinese medicinal plants, initially from southern regions, have been domesticated in northern China to meet growing demand. For example, *Lonicera japonica* has been cultivated on over 155 800 acres in regions like Hebei and Xinjiang (Huang and Zhang, 2020). This shift from warm to colder areas leads to changes in its leaf color and composition, including altered anthocyanin, chlorophyll, and carotenoid contents (Zhang et al., 2022b). Other plants, such as *Chrysanthemum morifolium*, have also undergone changes in response to colder conditions, accumulating cyanidin and pelargonidin derivatives and shifting from white to purple petals (Wu et al., 2024b). In addition, *Andrographis paniculata*, introduced to southern China and later cultivated in the north, has developed novel labdane-type diterpenoids with anti-inflammatory properties (Yu et al., 2023). These independent domestication events often exhibit a domestication syndrome, shaped by human selection, which we describe as a "short-term anthropogenic experiment in convergent evolution." As genomic selection and molecular breeding advance, convergent selection for desirable traits and functions beyond cold adaptation is likely to increase.

CLIMATE ZONE SHIFTS AND PLANT MIGRATION OR ADAPTATION

Shifts in frost, chilling, and warm zones under global warming

Global warming is widely recognized as an ongoing issue. Under various warming scenarios (Riahi et al., 2017; Zhu et al., 2023), our projections indicate a significant reduction in frost zones by 2081–2100, with a decline of 4%–11% (Figure 6). By contrast, warm zones are expected to expand by 2%–12%. Chilling zones are expected to maintain their overall size owing to a balance between influx and outflow from frost zones and warm zones. However, plants situated in transitional areas will experience significant impacts. These changes are contingent upon anthropogenic warming scenarios, which range from sustainable low emissions (SSP126) to severe climate change fueled by rapid growth and heavy fossil fuel dependence (SSP585) (Riahi et al., 2017; Zhu et al., 2023). The projections are based on idealized models but could underrepresent real-world complexities. For example, with rising global temperatures, the size of forest fires in Canada, the USA, and Australia has doubled or even tripled in recent decades (Zhao et al., 2024).

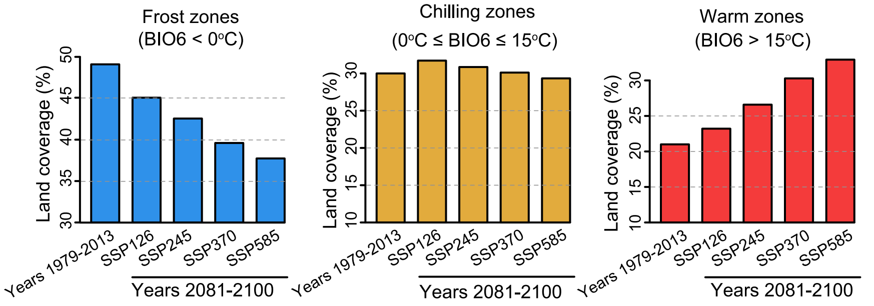

Figure 6. Projection of frost, chilling, and warm zones under global warming.
The projection is based on the extent of different warming scenarios, ranging from low to high radiative forcing: SSP126, SSP245, SSP370, and SSP585 (Riahi et al., 2017; Zhu et al., 2023). The temperature estimate (BIO6) for 2081–2100 on the UKESM1-0-LL model, sourced from WorldClim Global Climate Data (Fick and Hijmans, 2017), was used in this analysis.

As warmer zones expand and frost zones diminish, more plants are subjected to higher temperatures, which can significantly affect their growth, flowering, fruiting, and overall ecosystem dynamics (Parmesan and Hanley, 2015). By contrast, the effect of low temperatures on plants is generally decreasing. However, this warming trend is not universally beneficial, as some plants require low-temperature accumulation to break dormancy or trigger flowering (Ye et al., 2021; Ding et al., 2024). Rising autumn temperatures can also lead to deacclimation and a loss of freezing tolerance, making plants vulnerable to freezing events (Vyse et al., 2019). In addition, milder winters can increase the survival rates of pests and pathogens, endangering plant health (Bebber et al., 2013). Despite the overall warming trend, changes in the melting of polar ice and atmospheric circulation patterns could trigger more frequent or severe extreme cold events, thereby imposing greater stress on plants (Francis and Vavrus, 2012; Cohen et al., 2014; Vitasse et al., 2018). As a result, plants may face more frequent exposures to unexpected frosts, and the reprogramming of gene expression for cold acclimation and freezing tolerance may become increasingly critical for their survival.

Niche conservation and/or niche evolution

In the context of expanding warmer zones and diminishing frost zones, the response of plant species, whether through migration or *in situ* adaptive evolution, is a complex issue (Donoghue and Edwards, 2014). On one hand, niche conservation suggests that plant species may prefer migration to maintain their ecological niches, as it might be "easier to move than to evolve" (Donoghue, 2008). This is supported by the fact that plants with effective seed dispersal mechanisms can migrate to suitable habitats as climate zones shift (Corlett and Westcott, 2013; Lenoir and Svenning, 2015). However, the rapid pace of climate change poses challenges to migration, as it can lead to habitat fragmentation and outpace the ability of many species to adapt, making it difficult for them to keep up (Corlett and Westcott, 2013; Urban, 2015).

On the other hand, *in situ* adaptive evolution may become a viable strategy for plants, particularly those with short generation times and high genetic diversity, enabling them to rapidly respond to environmental changes (Edelsparre et al., 2024). Climate change can impose selective pressures, such as increased temperatures and altered precipitation patterns, likely driving the evolution of new traits that enhance survival in existing habitats (Hoffmann and Sgrò, 2011). Gene flow and hybridization can also introduce new genetic variations, facilitating adaptation (Hamilton and Miller, 2016). Recent evidence suggests that some plant species are already showing signs of rapid adaptive evolution in response to climate change (Exposito-Alonso et al., 2018). Ultimately, plant species will likely employ a combination of migration and evolution to respond to climate change, influenced by their biological characteristics, ecological requirements, and the specific environmental challenges they face.

CONCLUDING REMARKS AND FUTURE DIRECTIONS

Spatiotemporal convergent evolution reveals insights into the adaptive strategies of angiosperms under environmental pressures. Convergent evolution illustrates the predictability and repeatability of evolutionary processes, driven by deterministic forces such as natural selection and genetic constraints (Stern, 2013; Bolnick et al., 2018; James et al., 2023). The independent evolution of similar traits or functions across diverse plant lineages highlights common evolutionary pressures that shape optimal solutions for survival in frost environments. The present review is intended to deepen our understanding of plant adaptations and offer a framework for predicting plant responses to future climatic changes.

Plants have developed a range of cold adaptation mechanisms, from visible traits such as herbaceous growth and leaf deciduousness to complex hidden molecular pathways. The exploration of convergent evolution reveals a fascinating array of molecular mechanisms that facilitate the adaptation of plants to environmental challenges. These mechanisms include amino acid substitutions, changes in gene duplicates and/or their expression, and alterations in different genes within the same or similar functional pathways (Stern, 2013; Wu et al., 2020b; James et al., 2023). These processes collectively enhance plant adaptability and resilience in frost regions. A notable example is cold acclimation, in which plants activate regulatory networks like the CBF/DREB1 pathway to improve freezing tolerance (Shi et al., 2018; Kidokoro et al., 2022; Ding et al., 2024). However, the effectiveness of these pathways varies among species, reflecting the complexity and diversity of plant responses to cold stress. Although identical genetic changes are rare across species, convergence occurs in functionally related genes within the same families and/or stress response pathways, highlighting shared evolutionary pressures that drive similar functions and adaptations (Schubert et al., 2019; Birkeland et al., 2020; Zhang et al., 2023). Gene duplications, particularly WGDs and TDs, play a key role in providing genetic material for innovation, enabling plants to improve their adaptations to

environmental pressures (Van de Peer et al., 2021; Guo et al., 2022, 2024; Wu et al., 2024a).

Looking forward, the increasing availability of genomic data and plant traits offers unprecedented opportunities to further reveal the complexities of genetic convergence. Future research should prioritize comparative studies to determine the relative importance of different modes of molecular convergence and their contributions to environmental adaptations. This includes examining the effects of positive selection, convergent site substitutions, and, in particular, changes in gene copy number and expression within functionally related pathways that drive the evolution of adaptive traits. It is also crucial to explore how gene flow and hybridization introduce novel genetic variations that facilitate adaptation (Hamilton and Miller, 2016). These insights are vital for predicting plant responses to climate change, conserving biodiversity, and developing resilient agricultural systems.

FUNDING
This work is supported by the National Natural Science Foundation of China (32470665), the Scientific and Technological Innovation Project of China Academy of Chinese Medical Sciences (CI2023E002), the Innovation Team and Talents Cultivation Program of the National Administration of Traditional Chinese Medicine (ZYYCXTD-D-202005 and ZYYZDXK-2023244), and the China Agriculture Research System of MOF and MARA (CARS-21).

ACKNOWLEDGMENTS
We apologize to colleagues whose work is not cited owing to space limitations and our limited expertise in this field. No conflict of interest is declared.

AUTHOR CONTRIBUTIONS
S.W., J.Y., and W.W. conceived and developed the idea. J.Y. and W.W. managed the project. S.W., J.L., P.Y., L.G., and J.Z. developed the methods, performed the analysis, and reviewed the relevant papers. S.W., J.L., P.Y., and W.W. drafted and polished the manuscript.

DECLARATION OF GENERATIVE AI AND AI-ASSISTED TECHNOLOGIES IN THE WRITING PROCESS
During the preparation of this work, the authors used ChatGPT to enhance the grammar, clarity, and readability of the text. After using this tool, the authors reviewed and edited the content as needed and take full responsibility for the content of the publication.

SUPPLEMENTAL INFORMATION
Supplemental information is available at *Plant Communications Online*.

Received: October 7, 2024
Revised: November 29, 2024
Accepted: January 18, 2025
Published: January 23, 2025

REFERENCES
Allen, D.J., and Ort, D.R. (2001). Impacts of chilling temperatures on photosynthesis in warm-climate plants. Trends Plant Sci. **6**:36–42.

Arendt, J., and Reznick, D. (2008). Convergence and parallelism reconsidered: what have we learned about the genetics of adaptation? Trends Ecol. Evol. **23**:26–32.

Augspurger, C.K., and Salk, C.F. (2017). Constraints of cold and shade on the phenology of spring ephemeral herb species. J. Ecol. **105**:246–254.

Axelrod, D.I. (1966). Origin of deciduous and evergreen habits in temperate forests. Evolution **20**:1–15.

Barrero-Gil, J., and Salinas, J. (2017). CBFs at the crossroads of plant hormone signaling in cold stress response. Mol. Plant **10**:542–544.

Barrett, S.C.H. (2002). The evolution of plant sexual diversity. Nat. Rev. Genet. **3**:274–284.

Barry, R.G. (2008). Mountain Weather and Climate (Cambridge University Press).

Bebber, D.P., Ramotowski, M.A.T., and Gurr, S.J. (2013). Crop pests and pathogens move polewards in a warming world. Nat. Clim. Change **3**:985–988.

Birkeland, S., Gustafsson, A.L.S., Brysting, A.K., Brochmann, C., and Nowak, M.D. (2020). Multiple genetic trajectories to extreme abiotic stress adaptation in Arctic Brassicaceae. Mol. Biol. Evol. **37**:2052–2068.

Birkeland, S., Slotte, T., Krag Brysting, A., Gustafsson, A.L.S., Rhoden Hvidsten, T., Brochmann, C., and Nowak, M.D. (2022). What can cold-induced transcriptomes of Arctic Brassicaceae tell us about the evolution of cold tolerance? Mol. Ecol. **31**:4271–4285.

Blount, Z.D., Lenski, R.E., and Losos, J.B. (2018). Contingency and determinism in evolution: Replaying life's tape. Science **362**:eaam5979.

Bolnick, D.I., Barrett, R.D., Oke, K.B., Rennison, D.J., and Stuart, Y.E. (2018). (Non)Parallel Evolution. Annu. Rev. Ecol. Evol. Syst. **49**:303–330.

Bowen, J.L., Kearns, P.J., Byrnes, J.E.K., Wigginton, S., Allen, W.J., Greenwood, M., Tran, K., Yu, J., Cronin, J.T., and Meyerson, L.A. (2017). Lineage overwhelms environmental conditions in determining rhizosphere bacterial community structure in a cosmopolitan invasive plant. Nat. Commun. **8**:433.

Bredow, M., and Walker, V.K. (2017). Ice-Binding Proteins in Plants. Front. Plant Sci. **8**:2153.

Brochmann, C., Brysting, A.K., Alsos, I.G., Borgen, L., Grundt, H.H., Scheen, A.C., and Elven, R. (2004). Polyploidy in arctic plants. Biol. J. Linn. Soc. Lond. **82**:521–536.

Carruthers, T., Gonçalves, D.J.P., Li, P., Chanderbali, A.S., Dick, C.W., Fritsch, P.W., Larson, D.A., Soltis, D.E., Soltis, P.S., Weaver, W.N., and Smith, S.A. (2024). Repeated shifts out of tropical climates preceded by whole genome duplication. New Phytol. **244**:2561–2575.

Cerca, J. (2023). Understanding natural selection and similarity: Convergent, parallel and repeated evolution. Mol. Ecol. **32**:5451–5462.

Chase, M.W., Christenhusz, M.J.M., Fay, M.F., Byng, J.W., Judd, W.S., Soltis, D.E., Mabberley, D.J., Sennikov, A.N., Soltis, P.S., Stevens, P.F., et al. (2016). An update of the Angiosperm Phylogeny Group classification for the orders and families of flowering plants: APG IV. Bot. J. Linn. Soc. **181**:1–20.

Chen, L., Luo, J., Jin, M., Yang, N., Liu, X., Peng, Y., Li, W., Phillips, A., Cameron, B., Bernal, J.S., et al. (2022). Genome sequencing reveals evidence of adaptive variation in the genus *Zea*. Nat. Genet. **54**:1736–1745.

Cheng, D., Liu, Y., Wang, Y., Cao, L., Wu, S., Yu, S., Xie, L.N., Li, H., Jiang, J., Liu, G., et al. (2024). Establishment of high-efficiency genome editing in white birch (*Betula platyphylla* Suk.). Plant Biotechnol. J. **22**:7–9.

Chik, W.I., Zhu, L., Fan, L.L., Yi, T., Zhu, G.Y., Gou, X.J., Tang, Y.N., Xu, J., Yeung, W.P., Zhao, Z.Z., et al. (2015). Saussurea involucrata: A review of the botany, phytochemistry and ethnopharmacology of a rare traditional herbal medicine. J. Ethnopharmacol. **172**:44–60.

Chinnusamy, V., Zhu, J., and Zhu, J.K. (2007). Cold stress regulation of gene expression in plants. Trends Plant Sci. **12**:444–451.

Christin, P.A., Salamin, N., Muasya, A.M., Roalson, E.H., Russier, F., and Besnard, G. (2008). Evolutionary switch and genetic convergence on rbcL following the evolution of C4 photosynthesis. Mol. Biol. Evol. **25**:2361–2368.

Christin, P.A., Salamin, N., Savolainen, V., Duvall, M.R., and Besnard, G. (2007). C4 photosynthesis evolved in grasses via parallel adaptive genetic changes. Curr. Biol. **17**:1241–1247.

Clifford, P., Richardson, S., and Hémon, D. (1989). Assessing the significance of the correlation between two spatial processes. Biometrics **45**:123–134.

Cohen, J., Screen, J.A., Furtado, J.C., Barlow, M., Whittleston, D., Coumou, D., Francis, J., Dethloff, K., Entekhabi, D., Overland, J., and Jones, J. (2014). Recent Arctic amplification and extreme mid-latitude weather. Nat. Geosci. **7**:627–637.

Comai, L. (2005). The advantages and disadvantages of being polyploid. Nat. Rev. Genet. **6**:836–846.

Corlett, R.T., and Westcott, D.A. (2013). Will plant movements keep up with climate change? Trends Ecol. Evol. **28**:482–488.

Cousins-Westerberg, R., Dakin, N., Schat, L., Kadereit, G., and Humphreys, A.M. (2023). Evolution of cold tolerance in the highly stress-tolerant samphires and relatives (Salicornieae: Amaranthaceae). Bot. J. Linn. Soc. **203**:20–36.

Crepet, W.L., and Niklas, K.J. (2009). Darwin's second 'abominable mystery': Why are there so many angiosperm species? Am. J. Bot. **96**:366–381.

Darwin, C. (1859). On the Origin of Species by Means of Natural Selection, or, the Preservation of Favoured Races in the Struggle for Life (London: John Murray).

Darwin, C. (1993). [The Correspondence]; the Correspondence of Charles Darwin (Cambridge University Press).

Davis, S.D., Sperry, J.S., and Hacke, U.G. (1999). The relationship between xylem conduit diameter and cavitation caused by freezing. Am. J. Bot. **86**:1367–1372.

Deng, B., Du, W., Liu, C., Sun, W., Tian, S., and Dong, H. (2012). Antioxidant response to drought, cold and nutrient stress in two ploidy levels of tobacco plants: low resource requirement confers polytolerance in polyploids? Plant Growth Regul. **66**:37–47.

Deng, D., Guo, Y., Guo, L., Li, C., Nie, Y., Wang, S., and Wu, W. (2024). Functional divergence in orthologous transcription factors: insights from AtCBF2/3/1 and OsDREB1C. Mol. Biol. Evol. **41**:msae089.

DeSalle, R. (2000). Adaptive evolution of genes and genomes. Heredity **85**:303.

Ding, Y., Shi, Y., and Yang, S. (2020). Molecular regulation of plant responses to environmental temperatures. Mol. Plant **13**:544–564.

Ding, Y., Shi, Y., and Yang, S. (2024). Regulatory networks underlying plant responses and adaptation to cold stress. Annu. Rev. Genet. **58**:43–65.

Doebley, J.F., Gaut, B.S., and Smith, B.D. (2006). The molecular genetics of crop domestication. Cell **127**:1309–1321.

Donoghue, M.J. (2008). A phylogenetic perspective on the distribution of plant diversity. Proc. Natl. Acad. Sci. USA **105**:11549–11555.

Donoghue, M.J., and Edwards, E.J. (2014). Biome shifts and niche evolution in plants. Annu. Rev. Ecol. Evol. Syst. **45**:547–572.

Dreyer, A., and Dietz, K.J. (2018). Reactive oxygen species and the redox-regulatory network in cold stress acclimation. Antioxidants **7**:169.

Edelsparre, A.H., Fitzpatrick, M.J., Saastamoinen, M., and Teplitsky, C. (2024). Evolutionary adaptation to climate change. Evol. Lett. **8**:1–7.

Edwards, E.J., Chatelet, D.S., Chen, B.C., Ong, J.Y., Tagane, S., Kanemitsu, H., Tagawa, K., Teramoto, K., Park, B., Chung, K.F., et al. (2017). Convergence, consilience, and the evolution of temperate deciduous forests. Am. Nat. **190**:S87–S104.

Edwards, E.J., de Vos, J.M., and Donoghue, M.J. (2015). Doubtful pathways to cold tolerance in plants. Nature **521**:E5–E6.

Exposito-Alonso, M., Vasseur, F., Ding, W., Wang, G., Burbano, H.A., and Weigel, D. (2018). Genomic basis and evolutionary potential for extreme drought adaptation in *Arabidopsis thaliana*. Nat. Ecol. Evol. **2**:352–358.

Eyre-Walker, A., and Keightley, P.D. (2007). The distribution of fitness effects of new mutations. Nat. Rev. Genet. **8**:610–618.

Fan, H.H., Wei, J., Li, T.C., Li, Z.P., Guo, N., Cai, Y.P., and Lin, Y. (2013). DNA methylation alterations of upland cotton (*Gossypium hirsutum*) in response to cold stress. Acta Physiol. Plant. **35**:2445–2453.

Feild, T.S., Arens, N.C., Doyle, J.A., Dawson, T.E., and Donoghue, M.J. (2004). Dark and disturbed: a new image of early angiosperm ecology. Paleobiology **30**:82–107.

Fick, S.E., and Hijmans, R.J. (2017). WorldClim 2: new 1-km spatial resolution climate surfaces for global land areas. Int. J. Climatol. **37**:4302–4315.

Folk, R.A., Siniscalchi, C.M., and Soltis, D.E. (2020). Angiosperms at the edge: Extremity, diversity, and phylogeny. Plant Cell Environ. **43**:2871–2893.

Foote, A.D., Liu, Y., Thomas, G.W.C., Vinař, T., Alföldi, J., Deng, J., Dugan, S., van Elk, C.E., Hunter, M.E., Joshi, V., et al. (2015). Convergent evolution of the genomes of marine mammals. Nat. Genet. **47**:272–275.

Force, A., Lynch, M., Pickett, F.B., Amores, A., Yan, Y.L., and Postlethwait, J. (1999). Preservation of duplicate genes by complementary, degenerative mutations. Genetics **151**:1531–1545.

Francis, J.A., and Vavrus, S.J. (2012). Evidence linking Arctic amplification to extreme weather in mid-latitudes. Geophys. Res. Lett. **39**:L06801.

Friedman, J., and Barrett, S.C.H. (2009). Wind of change: new insights on the ecology and evolution of pollination and mating in wind-pollinated plants. Ann. Bot. **103**:1515–1527.

Gale, J. (2004). Plants and altitude - revisited. Ann. Bot. **94**:199.

González-Zurdo, P., Escudero, A., Babiano, J., García-Ciudad, A., and Mediavilla, S. (2016). Costs of leaf reinforcement in response to winter cold in evergreen species. Tree Physiol. **36**:273–286.

Gould, S.J. (1989). Wonderful Life (WW Norton & Company).

Groover, A.T. (2005). What genes make a tree a tree? Trends Plant Sci. **10**:210–214.

Guo, L., Wang, S., Jiao, X., Ye, X., Deng, D., Liu, H., Li, Y., Van de Peer, Y., and Wu, W. (2024). Convergent and/or parallel evolution of RNA-binding proteins in angiosperms after polyploidization. New Phytol. **242**:1377–1393.

Guo, L., Wang, S., Nie, Y., Shen, Y., Ye, X., and Wu, W. (2022). Convergent evolution of AP2/ERF III and IX subfamilies through recurrent polyploidization and tandem duplication during eudicot adaptation to paleoenvironmental changes. Plant Commun. **3**:100420.

Guo, L., Xu, Z., Wang, S., Nie, Y., Ye, X., Jin, X., Zhu, J., and Wu, W. (2023). Integrative multi-omics analysis of three early diverged rosid species reveals an ancient hierarchical cold-responsive regulatory network. Physiol. Plantarum **175**:e13892.

Gupta, R., and Deswal, R. (2014). Antifreeze proteins enable plants to survive in freezing conditions. J. Bio. Sci. **39**:931–944.

Gutschick, V.P., and BassiriRad, H. (2003). Extreme events as shaping physiology, ecology, and evolution of plants: toward a unified definition and evaluation of their consequences. New Phytol. **160**:21–42.

Halbritter, A.H., Fior, S., Keller, I., Billeter, R., Edwards, P.J., Holderegger, R., Karrenberg, S., Pluess, A.R., Widmer, A., and Alexander, J.M. (2018). Trait differentiation and adaptation of plants along elevation gradients. J. Evol. Biol. **31**:784–800.

Hamilton, J.A., and Miller, J.M. (2016). Adaptive introgression as a resource for management and genetic conservation in a changing climate. Conserv. Biol. **30**:33–41.

Hao, Y., Wang, X.F., Guo, Y., Li, T.Y., Yang, J., Ainouche, M.L., Salmon, A., Ju, R.T., Wu, J.H., Li, L.F., and Li, B. (2024). Genomic and phenotypic signatures provide insights into the wide adaptation of a global plant invader. Plant Commun. **5**:100820.

He, Z., Feng, X., Chen, Q., Li, L., Li, S., Han, K., Guo, Z., Wang, J., Liu, M., Shi, C., et al. (2022). Evolution of coastal forests based on a full set of mangrove genomes. Nat. Ecol. Evol. **6**:738–749.

He, Z., Xu, S., Zhang, Z., Guo, W., Lyu, H., Zhong, C., Boufford, D.E., Duke, N.C., and International Mangrove Consortium, and Shi, S. (2020). Convergent adaptation of the genomes of woody plants at the land-sea interface. Natl. Sci. Rev. **7**:978–993.

Heyduk, K., Moreno-Villena, J.J., Gilman, I.S., Christin, P.A., and Edwards, E.J. (2019). The genetics of convergent evolution: insights from plant photosynthesis. Nat. Rev. Genet. **20**:485–493.

Hjertaas, A.C., Preston, J.C., Kainulainen, K., Humphreys, A.M., and Fjellheim, S. (2023). Convergent evolution of the annual life history syndrome from perennial ancestors. Front. Plant Sci. **13**:1048656.

Hoffmann, A.A., and Sgrò, C.M. (2011). Climate change and evolutionary adaptation. Nature **470**:479–485.

Huang, L., and Zhang, X. (2020). Statistical Report on the Production of Chinese Herbal Medicines in China (Shanghai Scientific & Technical Publishers).

James, M.E., Brodribb, T., Wright, I.J., Rieseberg, L.H., and Ortiz-Barrientos, D. (2023). Replicated Evolution in Plants. Annu. Rev. Plant Biol. **74**:697–725.

Jia, Y., Ding, Y., Shi, Y., Zhang, X., Gong, Z., and Yang, S. (2016). The *cbfs* triple mutants reveal the essential functions of CBFs in cold acclimation and allow the definition of CBF regulons in *Arabidopsis*. New Phytol. **212**:345–353.

Jiang, Y., Liu, S., Hu, J., He, G., Liu, Y., Chen, X., Lei, T., Li, Q., Yang, L., Li, W., et al. (2020). Polyploidization of *Plumbago auriculata* Lam. in vitro and its characterization including cold tolerance. Plant Cell Tissue Organ Cult. **140**:315–325.

Jin, Y., and Qian, H. (2022). V.PhyloMaker2: An updated and enlarged R package that can generate very large phylogenies for vascular plants. Plant Divers. **44**:335–339.

Karger, D.N., Conrad, O., Böhner, J., Kawohl, T., Kreft, H., Soria-Auza, R.W., Zimmermann, N.E., Linder, H.P., and Kessler, M. (2017). Climatologies at high resolution for the earth's land surface areas. Sci. Data **4**:170122.

Kerbler, S.M., and Wigge, P.A. (2023). Temperature sensing in plants. Annu. Rev. Plant Biol. **74**:341–366.

Kerkhoff, A.J., Moriarty, P.E., and Weiser, M.D. (2014). The latitudinal species richness gradient in New World woody angiosperms is consistent with the tropical conservatism hypothesis. Proc. Natl. Acad. Sci. USA **111**:8125–8130.

Kevan, P.G. (1972). Insect pollination of high arctic flowers. J. Ecol. **60**:831–847.

Kidokoro, S., Shinozaki, K., and Yamaguchi-Shinozaki, K. (2022). Transcriptional regulatory network of plant cold-stress responses. Trends Plant Sci. **27**:922–935.

Klimeš, A., Šímová, I., Zizka, A., Antonelli, A., and Herben, T. (2022). The ecological drivers of growth form evolution in flowering plants. J. Ecol. **110**:1525–1536.

Körner, C. (2021). Alpine Plant Life: Functional Plant Ecology of High Mountain Ecosystems (Springer Nature).

Körner, C., Neumayer, M., Menendez-Riedl, S.P., and Smeets-Scheel, A. (1989). Functional morphology of mountain plants. Flora **182**:353–383.

Kosová, K., Prášil, I.T., and Vítámvás, P. (2019). Role of dehydrins in plant stress response. In Handbook of Plant and Crop Stress, fourth edition (CRC Press), pp. 175–196.

Lancaster, L.T., and Humphreys, A.M. (2020). Global variation in the thermal tolerances of plants. Proc. Natl. Acad. Sci. USA **117**:13580–13587.

Lenoir, J., and Svenning, J.C. (2015). Climate-related range shifts - a global multidimensional synthesis and new research directions. Ecography **38**:15–28.

Levitt, J. (1980). Responses of Plants to Environmental Stress, Volume 1: Chilling, Freezing, and High Temperature Stresses (Academic Press).

Liu, B., Zhao, F.M., Cao, Y., Wang, X.Y., Li, Z., Shentu, Y., Zhou, H., and Xia, Y.P. (2022). Photoprotection contributes to freezing tolerance as revealed by RNA-seq profiling of Rhododendron leaves during cold acclimation and deacclimation over time. Hortic. Res. **9**:uhab025.

Liu, W., Zheng, L., and Qi, D. (2020). Variation in leaf traits at different altitudes reflects the adaptive strategy of plants to environmental changes. Ecol. Evol. **10**:8166–8175.

Liu, Y., Xu, X., Dimitrov, D., Pellissier, L., Borregaard, M.K., Shrestha, N., Su, X., Luo, A., Zimmermann, N.E., Rahbek, C., and Wang, Z. (2023). An updated floristic map of the world. Nat. Commun. **14**:2990.

Lu, L.M., Mao, L.F., Yang, T., Ye, J.F., Liu, B., Li, H.L., Sun, M., Miller, J.T., Mathews, S., Hu, H.H., et al. (2018). Evolutionary history of the angiosperm flora of China. Nature **554**:234–238.

Lynch, M., and Conery, J.S. (2000). The evolutionary fate and consequences of duplicate genes. Science **290**:1151–1155.

Ma, H., Crowther, T.W., Mo, L., Maynard, D.S., Renner, S.S., van den Hoogen, J., Zou, Y., Liang, J., De-Miguel, S., Nabuurs, G.J., et al. (2023). The global biogeography of tree leaf form and habit. Nat. Plants **9**:1795–1809.

Ma, Y., Dai, X., Xu, Y., Luo, W., Zheng, X., Zeng, D., Pan, Y., Lin, X., Liu, H., Zhang, D., et al. (2015). COLD1 confers chilling tolerance in rice. Cell **160**:1209–1221.

Mahler, D.L., Ingram, T., Revell, L.J., and Losos, J.B. (2013). Exceptional convergence on the macroevolutionary landscape in island lizard radiations. Science **341**:292–295.

Martin, S.L., and Husband, B.C. (2009). Influence of phylogeny and ploidy on species ranges of North American angiosperms. J. Ecol. **97**:913–922.

Maruyama, K., Todaka, D., Mizoi, J., Yoshida, T., Kidokoro, S., Matsukura, S., Takasaki, H., Sakurai, T., Yamamoto, Y.Y., Yoshiwara, K., et al. (2012). Identification of *cis*-acting promoter elements in cold- and dehydration-induced transcriptional pathways in *Arabidopsis*, rice, and soybean. DNA Res. **19**:37–49.

Massante, J.C., Götzenberger, L., Takkis, K., Hallikma, T., Kaasik, A., Laanisto, L., Hutchings, M.J., and Gerhold, P. (2019). Contrasting latitudinal patterns in phylogenetic diversity between woody and herbaceous communities. Sci. Rep. **9**:6443.

McGhee, G.R. (2011). Convergent Evolution: Limited Forms Most Beautiful (MIT press).

Moles, A.T., Warton, D.I., Warman, L., Swenson, N.G., Laffan, S.W., Zanne, A.E., Pitman, A., Hemmings, F.A., and Leishman, M.R. (2009). Global patterns in plant height. J. Ecol. **97**:923–932.

Morris, S.C. (1998). The Crucible of Creation: The Burgess Shale and the Rise of Animals (Oxford University Press).

Morris, S.C. (2003). Life's Solution (Cambridge university press).

Murat, F., Van de Peer, Y., and Salse, J. (2012). Decoding plant and animal genome plasticity from differential paleo-evolutionary patterns and processes. Genome Biol. Evol. **4**:917–928.

Murphy, P.G., and Lugo, A.E. (1986). Ecology of tropical dry forest. Annu. Rev. Ecol. Systemat. **17**:67–88.

Nie, Y., Guo, L., Cui, F., Shen, Y., Ye, X., Deng, D., Wang, S., Zhu, J., and Wu, W. (2022). Innovations and stepwise evolution of CBFs/ DREB1s and their regulatory networks in angiosperms. J. Integr. Plant Biol. **64**:2111–2125.

Nievola, C.C., Carvalho, C.P., Carvalho, V., and Rodrigues, E. (2017). Rapid responses of plants to temperature changes. Temperature **4**:371–405.

Nürk, N.M., Atchison, G.W., and Hughes, C.E. (2019). Island woodiness underpins accelerated disparification in plant radiations. New Phytol. **224**:518–531.

Olson, D.M., Dinerstein, E., Wikramanayake, E.D., Burgess, N.D., Powell, G.V.N., Underwood, E.C., D'Amico, J.A., Itoua, I., Strand, H.E., Morrison, J.C., et al. (2001). Terrestrial Ecoregions of the World: A New Map of Life on Earth: A new global map of terrestrial ecoregions provides an innovative tool for conserving biodiversity. Bioscience **51**:933–938.

Orgogozo, V. (2015). Replaying the tape of life in the twenty-first century. Interface Focus **5**:20150057.

Park, S., Lee, C.M., Doherty, C.J., Gilmour, S.J., Kim, Y., and Thomashow, M.F. (2015). Regulation of the *Arabidopsis* CBF regulon by a complex low-temperature regulatory network. Plant J. **82**:193–207.

Parmesan, C., and Hanley, M.E. (2015). Plants and climate change: complexities and surprises. Ann. Bot. **116**:849–864.

Pearce, R. (2001). Plant freezing and damage. Ann. Bot. **87**:417–424.

Powell, R. (2020). Contingency and Convergence: Toward a Cosmic Biology of Body and Mind (MIT Press).

Powell, R., and Mariscal, C. (2015). Convergent evolution as natural experiment: the tape of life reconsidered. Interface Focus **5**:20150040.

Preston, J.C., and Sandve, S.R. (2013). Adaptation to seasonality and the winter freeze. Front. Plant Sci. **4**:167.

Rainey, P.B., and Travisano, M. (1998). Adaptive radiation in a heterogeneous environment. Nature **394**:69–72.

Rellstab, C., Zoller, S., Sailer, C., Tedder, A., Gugerli, F., Shimizu, K.K., Holderegger, R., Widmer, A., and Fischer, M.C. (2020). Genomic signatures of convergent adaptation to Alpine environments in three Brassicaceae species. Mol. Ecol. **29**:4350–4365.

Riahi, K., van Vuuren, D.P., Kriegler, E., Edmonds, J., O'Neill, B.C., Fujimori, S., Bauer, N., Calvin, K., Dellink, R., Fricko, O., et al. (2017). The Shared Socioeconomic Pathways and their energy, land use, and greenhouse gas emissions implications: An overview. Global Environ. Change **42**:153–168.

Rice, A., Šmarda, P., Novosolov, M., Drori, M., Glick, L., Sabath, N., Meiri, S., Belmaker, J., and Mayrose, I. (2019). The global biogeography of polyploid plants. Nat. Ecol. Evol. **3**:265–273.

Ricklefs, R.E., and Renner, S.S. (1994). Species richness within families of flowering plants. Evolution **48**:1619–1636.

Rodger, J.G., Bennett, J.M., Razanajatovo, M., Knight, T.M., van Kleunen, M., Ashman, T.L., Steets, J.A., Hui, C., Arceo-Gómez, G., Burd, M., et al. (2021). Widespread vulnerability of flowering plant seed production to pollinator declines. Sci. Adv. **7**:eabd3524.

Rosenblum, E.B., Parent, C.E., and Brandt, E.E. (2014). The molecular basis of phenotypic convergence. Annu. Rev. Ecol. Evol. Syst. **45**:203–226.

Sage, R.F., Christin, P.A., and Edwards, E.J. (2011). The C(4) plant lineages of planet Earth. J. Exp. Bot. **62**:3155–3169.

Sakai, A., and Larcher, W. (1987). Frost survival of plants: responses and adaptation to freezing stress. Ecol. Stud. **62**:321.

Salojärvi, J., Smolander, O.P., Nieminen, K., Rajaraman, S., Safronov, O., Safdari, P., Lamminmaki, A., Immanen, J., Lan, T., Tanskanen, J., et al. (2017). Genome sequencing and population genomic analyses provide insights into the adaptive landscape of silver birch. Nat. Genet. **49**:904–912.

Saxena, S.C., Kaur, H., Verma, P., Petla, B.P., Andugula, V.R., and Majee, M. (2013). Osmoprotectants: Potential for crop improvement under adverse conditions. In Plant Acclimation to Environmental Stress–Tuteja, S. Singh Gill, ed. (Springer New York), pp. 197–232.

Schinkel, C.C.F., Kirchheimer, B., Dellinger, A.S., Klatt, S., Winkler, M., Dullinger, S., and Hörandl, E. (2016). Correlations of polyploidy and apomixis with elevation and associated environmental gradients in an alpine plant. AoB Plants **8**:plw064.

Schluter, D. (1996). Ecological causes of adaptive radiation. Am. Nat. **148**:S40–S64.

Schubert, M., Grønvold, L., Sandve, S.R., Hvidsten, T.R., and Fjellheim, S. (2019). Evolution of cold acclimation and its role in niche transition in the temperate grass subfamily Pooideae. Plant Physiol. **180**:404–419.

Schubert, M., Humphreys, A.M., Lindberg, C.L., Preston, J.C., and Fjellheim, S. (2020). To coldly go where no grass has gone before: a multidisciplinary review of cold adaptation in Poaceae. Annual Plant Reviews Online **3**:523–562.

Schulte, P., Alegret, L., Arenillas, I., Arz, J.A., Barton, P.J., Bown, P.R., Bralower, T.J., Christeson, G.L., Claeys, P., Cockell, C.S., et al. (2010). The Chicxulub asteroid impact and mass extinction at the Cretaceous-Paleogene boundary. Science **327**:1214–1218.

Scotese, C.R., Song, H., Mills, B.J., and van der Meer, D.G. (2021). Phanerozoic paleotemperatures: The earth's changing climate during the last 540 million years. Earth Sci. Rev. **215**:103503.

Shi, Y., Ding, Y., and Yang, S. (2018). Molecular regulation of CBF signaling in cold acclimation. Trends Plant Sci. **23**:623–637.

Smith, S.A., and Donoghue, M.J. (2008). Rates of molecular evolution are linked to life history in flowering plants. Science **322**:86–89.

Soltis, D.E., Bell, C.D., Kim, S., and Soltis, P.S. (2008). Origin and early evolution of angiosperms. Ann. N. Y. Acad. Sci. **1133**:3–25.

Song, X.M., Wang, J.P., Sun, P.C., Ma, X., Yang, Q.H., Hu, J.J., Sun, S.R., Li, Y.X., Yu, J.G., Feng, S.Y., et al. (2020). Preferential gene retention increases the robustness of cold regulation in Brassicaceae and other plants after polyploidization. Hortic. Res. **7**:20.

Song, Y., Zhang, X., Li, M., Yang, H., Fu, D., Lv, J., Ding, Y., Gong, Z., Shi, Y., and Yang, S. (2021). The direct targets of CBFs: in cold stress response and beyond. J. Integr. Plant Biol. **63**:1874–1887.

Spriggs, E.L., Clement, W.L., Sweeney, P.W., Madriñán, S., Edwards, E.J., and Donoghue, M.J. (2015). Temperate radiations and dying embers of a tropical past: the diversification of Viburnum. New Phytol. **207**:340–354.

Stern, D.L. (2013). The genetic causes of convergent evolution. Nat. Rev. Genet. **14**:751–764.

Storey, J.D., and Tibshirani, R. (2003). Statistical significance for genomewide studies. Proc. Natl. Acad. Sci. USA **100**:9440–9445.

Sugiyama, S. (1998). Differentiation in competitive ability and cold tolerance between diploid and tetraploid cultivars in *Lolium perenne*. Euphytica **103**:55–59.

Tang, X., Wang, Q., Yuan, H., and Huang, X. (2018). Chilling-induced DNA Demethylation is associated with the cold tolerance of *Hevea brasiliensis*. BMC Plant Biol. **18**:70.

Thomashow, M.F. (1999). Plant cold acclimation: freezing tolerance genes and regulatory mechanisms. Annu. Rev. Plant Biol. **50**:571–599.

Urban, M.C. (2015). Accelerating extinction risk from climate change. Science **348**:571–573.

Van de Peer, Y., Ashman, T.L., Soltis, P.S., and Soltis, D.E. (2021). Polyploidy: an evolutionary and ecological force in stressful times. Plant Cell **33**:11–26.

Vanneste, K., Baele, G., Maere, S., and Van de Peer, Y. (2014). Analysis of 41 plant genomes supports a wave of successful genome duplications in association with the Cretaceous-Paleogene boundary. Genome Res. **24**:1334–1347.

Vitasse, Y., Lenz, A., and Körner, C. (2014). The interaction between freezing tolerance and phenology in temperate deciduous trees. Front. Plant Sci. **5**:541.

Vitasse, Y., Schneider, L., Rixen, C., Christen, D., and Rebetez, M. (2018). Increase in the risk of exposure of forest and fruit trees to spring frosts at higher elevations in Switzerland over the last four decades. Agric. For. Meteorol. **248**:60–69.

Vitti, J.J., Grossman, S.R., and Sabeti, P.C. (2013). Detecting natural selection in genomic data. Annu. Rev. Genet. **47**:97–120.

Vyse, K., Pagter, M., Zuther, E., and Hincha, D.K. (2019). Deacclimation after cold acclimation-a crucial, but widely neglected part of plant winter survival. J. Exp. Bot. **70**:4595–4604.

Wang, S., Zhang, Y., Ye, X., Shen, Y., Liu, H., Zhao, X., Guo, L., Cao, L., Du, Y., and Wu, W. (2023a). A phylotranscriptomic dataset of angiosperm species under cold stress. Sci. Data **10**:399.

Wang, S., Shen, Y., Deng, D., Guo, L., Zhang, Y., Nie, Y., Du, Y., Zhao, X., Ye, X., Huang, J., et al. (2023b). Orthogroup and phylotranscriptomic analyses identify transcription factors involved in the plant cold response: A case study of *Arabidopsis* BBX29. Plant Commun. **4**:100684.

Wei, S., Li, X., Lu, Z., Zhang, H., Ye, X., Zhou, Y., Li, J., Yan, Y., Pei, H., Duan, F., et al. (2022). A transcriptional regulator that boosts grain yields and shortens the growth duration of rice. Science **377**:eabi8455.

Weigelt, P., König, C., and Kreft, H. (2020). GIFT - A Global Inventory of Floras and Traits for macroecology and biogeography. J. Biogeogr. **47**:16–43.

Whitmore, T.C. (1990). An Introduction to Tropical Rain Forests (Oxford University Press).

Wisniewski, M., Nassuth, A., and Arora, R. (2018). Cold hardiness in trees: A mini-review. Front. Plant Sci. **9**:1394.

Wolfe, J.A., and Upchurch, G.R. (1986). Vegetation, climatic and floral changes at the Cretaceous-Tertiary boundary. Nature **324**:148–152.

Wright, S.I., Kalisz, S., and Slotte, T. (2013). Evolutionary consequences of self-fertilization in plants. Proc. Biol. Sci. **280**:20130133.

Wu, S., Han, B., and Jiao, Y. (2020a). Genetic contribution of paleopolyploidy to adaptive evolution in angiosperms. Mol. Plant **13**:59–71.

Wu, C.I., Wang, G.D., and Xu, S. (2020b). Convergent adaptive evolution—how common, or how rare? Natl. Sci. Rev. **7**:945–946.

Wu, W., Guo, L., Yin, L., Cai, B., Li, J., Li, X., Yang, J., Zhou, H., Tao, Z., and Li, Y. (2024a). Genomic convergence in terrestrial root plants through tandem duplication in response to soil microbial pressures. Cell Rep. **43**.

Wu, D., Wu, Y., Gao, R., Zhang, Y., Zheng, R., Fang, M., Li, Y., Zhang, Y., Guan, L., and Gao, Y. (2024b). Integrated metabolomics and transcriptomics reveal the key role of flavonoids in the cold tolerance of chrysanthemum. Int. J. Mol. Sci. **25**:7589.

Xu, S., He, Z., Guo, Z., Zhang, Z., Wyckoff, G.J., Greenberg, A., Wu, C.I., and Shi, S. (2017). Genome-wide convergence during evolution of mangroves from woody plants. Mol. Biol. Evol. **34**:1008–1015.

Xu, S., Wang, J., Guo, Z., He, Z., and Shi, S. (2020). Genomic convergence in the adaptation to extreme environments. Plant Commun. **1**:100117.

Ye, X., Zhao, X., Sun, Y., Zhang, M., Feng, S., Zhou, A., Wu, W., Ma, S., and Liu, S. (2021). The underlying molecular conservation and diversification of dioecious flower and leaf buds provide insights into the development, dormancy breaking, flowering, and sex association of willows. Plant Physiol. Biochem. **167**:651–664.

Yu, Y., Wang, Y., Wang, G.C., Tan, C.Y., Wang, Y., Liu, J.S., and Wang, G.K. (2023). Andropanilides A-C, the novel labdane-type diterpenoids from *Andrographis paniculata* and their anti-inflammation activity. Nat. Prod. Bioprospect. **13**:31.

Zanne, A.E., Pearse, W.D., Cornwell, W.K., McGlinn, D.J., Wright, I.J., and Uyeda, J.C. (2018). Functional biogeography of angiosperms: life at the extremes. New Phytol. **218**:1697–1709.

Zanne, A.E., Tank, D.C., Cornwell, W.K., Eastman, J.M., Smith, S.A., FitzJohn, R.G., McGlinn, D.J., O'Meara, B.C., Moles, A.T., Reich, P.B., et al. (2014). Three keys to the radiation of angiosperms into freezing environments. Nature **506**:89–92.

Zhang, D., Ye, J., and Sun, H. (2016). Quantitative approaches to identify floristic units and centres of species endemism in the Qinghai-Tibetan Plateau, south-western China. J. Biogeogr. **43**:2465–2476.

Zhang, H., Zhu, J., Gong, Z., and Zhu, J.K. (2022a). Abiotic stress responses in plants. Nat. Rev. Genet. **23**:104–119.

Zhang, M., Li, M., Fu, H., Wang, K., Tian, X., Qiu, R., Liu, J., Gao, S., Zhong, Z., Yang, B., and Zhang, L. (2022b). Transcriptomic analysis reveals the molecular response of *Lonicera japonica* leaves to chilling stress. Front. Plant Sci. **13**:1092857.

Zhang, L., Wu, S., Chang, X., Wang, X., Zhao, Y., Xia, Y., Trigiano, R.N., Jiao, Y., and Chen, F. (2020). The ancient wave of polyploidization events in flowering plants and their facilitated adaptation to environmental stress. Plant Cell Environ. **43**:2847–2856.

Zhang, X., Kuang, T., Dong, W., Qian, Z., Zhang, H., Landis, J.B., Feng, T., Li, L., Sun, Y., Huang, J., et al. (2023). Genomic convergence underlying high-altitude adaptation in alpine plants. J. Integr. Plant Biol. **65**:1620–1635.

Zhao, C., Zhang, Z., Xie, S., Si, T., Li, Y., and Zhu, J.K. (2016). Mutational evidence for the critical role of CBF transcription factors in cold acclimation in *Arabidopsis*. Plant Physiol. **171**:2744–2759.

Zhao, J., Yue, C., Wang, J., Hantson, S., Wang, X., He, B., Li, G., Wang, L., Zhao, H., and Luyssaert, S. (2024). Forest fire size amplifies postfire land surface warming. Nature **633**:828–834.

Zhu, B., Cheng, Y., Hu, X., Chai, Y., Berghuijs, W.R., Borthwick, A.G.L., and Slater, L. (2023). Constrained tropical land temperature-precipitation sensitivity reveals decreasing evapotranspiration and faster vegetation greening in CMIP6 projections. npj Clim. Atmos. Sci. **6**:91.

Zizka, A., Onstein, R.E., Rozzi, R., Weigelt, P., Kreft, H., Steinbauer, M.J., Bruelheide, H., and Lens, F. (2022). The evolution of insular woodiness. Proc. Natl. Acad. Sci. USA **119**:e2208629119.

Plant Communications, Volume *6*

Supplemental information

Convergent evolution in angiosperms adapted to cold climates

Shuo Wang, Jing Li, Ping Yu, Liangyu Guo, Junhui Zhou, Jian Yang, and Wenwu Wu

Supplementary materials

Cold climate-driven convergent evolution among angiosperms

Shuo Wang[1,6], Jing Li[1,6], Ping Yu[2,3,6], Liangyu Guo[1], Junhui Zhou[2,3], Jian Yang[2,3*], Wenwu Wu[1,4,5*]

[1]State Key Laboratory of Subtropical Silviculture, College of Forestry and Biotechnology, Zhejiang A&F University; Hangzhou 311300, China.

[2]State Key Laboratory for Quality Ensurance and Sustainable Use of Dao-di Herbs, National Resource Center for Chinese Materia Medica, China Academy of Chinese Medical Sciences; Beijing 100700, China

[3]Evaluation and Research Center of Daodi Herbs of Jiangxi Province, Ganjiang New District, 330000, China

[4]Zhejiang International Science and Technology Cooperation Base for Plant Germplasm Resources Conservation and Utilization, Zhejiang A&F University; Hangzhou 311300, China

[5]Provincial Key Laboratory for Non-wood Forest and Quality Control and Utilization of Its Products, Zhejiang A&F University, Hangzhou 311300, China

[6]These authors contributed equally to this work

***Email:** yangchem2012@163.com; wwwu@zafu.edu.cn

This file includes:

Figure S1 to S2

References

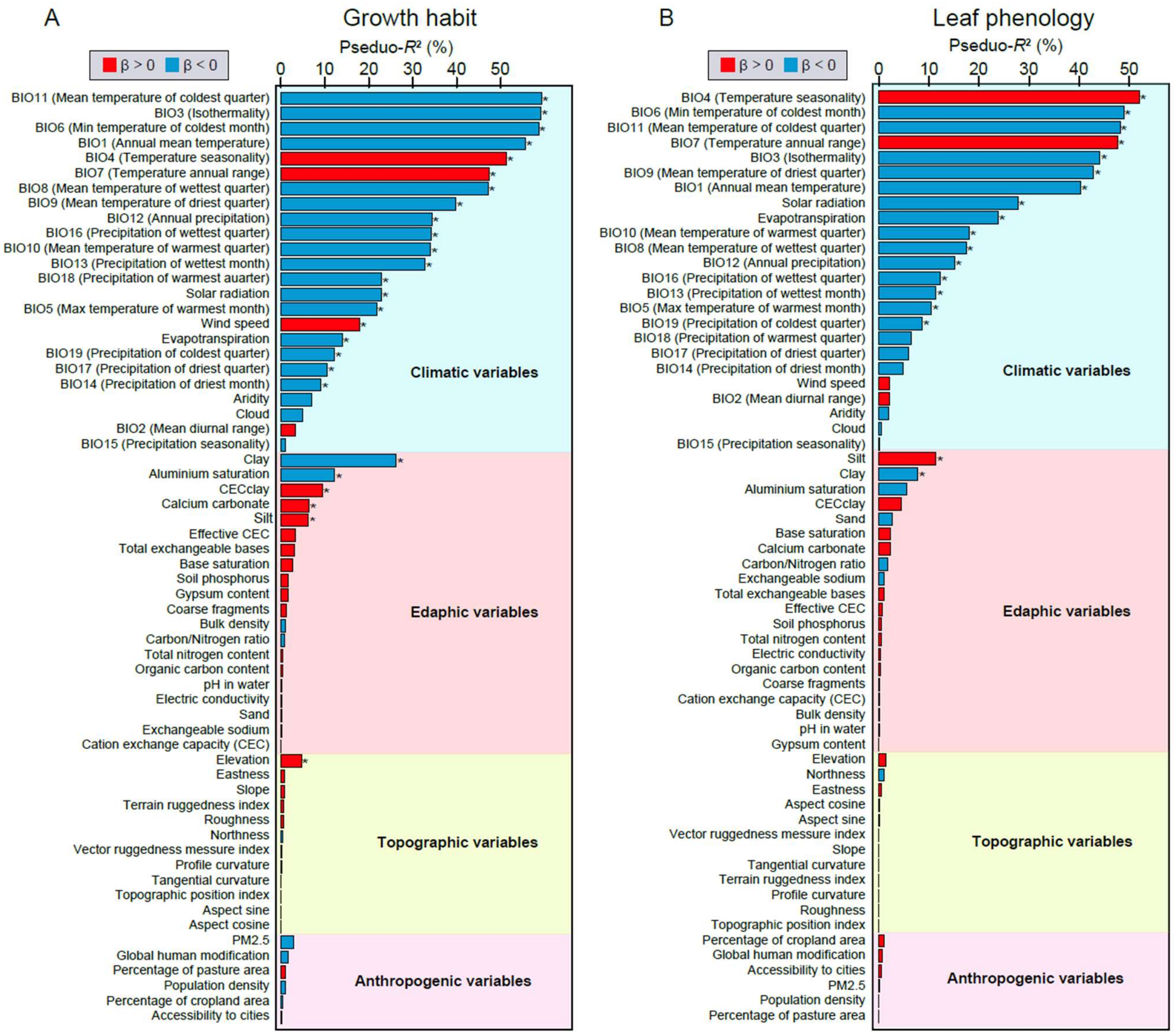

Supplementary Figure 1. Associations between geoenvironmental variables and plant traits. Beta regression was used to assess the association between each geoenvironmental variable (n = 62) and the frequency of herbaceous (**A**) and deciduous plants (**B**) across global ecoregions. The bar height represents the explained variance (pseudo-R^2), with positive effects ($\beta > 0$) indicated in red and negative effects ($\beta < 0$) in blue. To address potential spatial autocorrelation, a modified *t*-test was employed to assess their significance, followed by multiple testing correction using the Benjamini-Hochberg method. Statistically significant associations ($P_{adj} < 0.01$) are denoted by asterisks (*). The variables analyzed were sourced from public databases, including 19 bioclimatic variables (BIO1-BIO19) (CHELSA: chelsa-climate.org) (Karger et al., 2017), cloud cover (EarthEnv: www.earthenv.org/cloud), evapotranspiration and aridity (Zomer et al., 2022), cropland and pasture area (EarthStat: www.earthstat.org), accessibility to cities (Resource Watch: resourcewatch.org), and PM2.5 (SEDAC: sedac.ciesin.columbia.edu). The sources of other geoenvironmental variables have been described in our previous study (Wu et al., 2024).

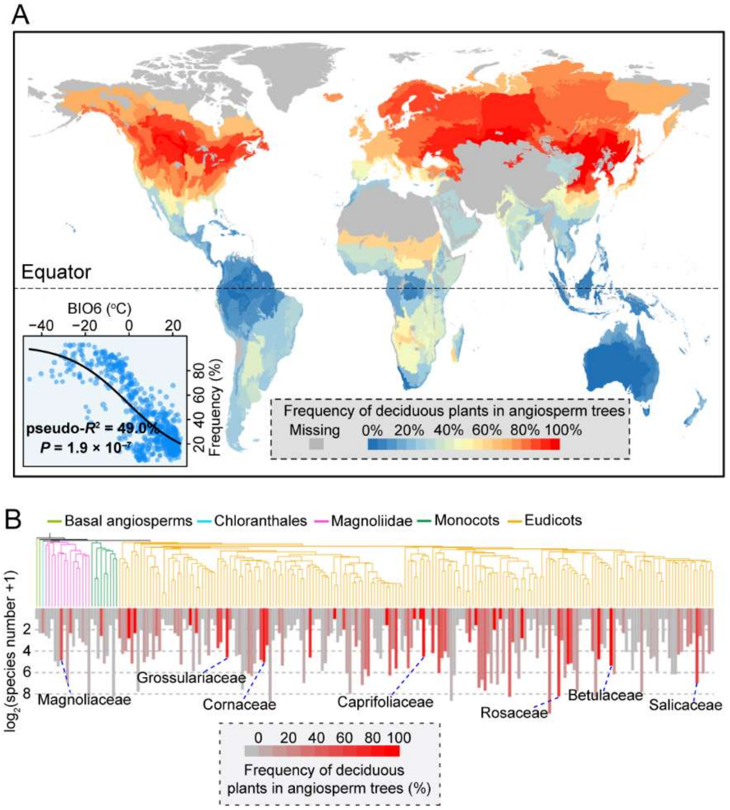

Supplementary Figure 2. Convergent evolution of leaf deciduousness in freezing temperatures. (**A**) An ecoregion-level map of the frequency of deciduous woody species. The frequency of deciduous woody plants at ecoregion level is defined as their proportion relative to the total number of deciduous and evergreen woody species collected within each ecoregion. Among the 55,557 woody angiosperm species (Fig 3A), many lacked deciduous or evergreen annotations. We supplemented these data with publicly available information (Weigelt et al., 2020; Zanne et al., 2014), yielding 2,086 deciduous and 6,757 evergreen woody plant species. At the lower left corner, we used the same method as Figure 3A to evaluate the association of deciduous frequency and BIO6.

(**B**) Independent evolution of leaf deciduousness across angiosperm woody species. Angiosperm families containing woody species were used to construct this phylogenetic tree. Below the angiosperm family tree, a bar graph shows the log2-transformed number of deciduous woody species per family in frost zones. The bars are shaded to indicate the proportion of deciduous woody species within each family that can inhabit frost zones, with darker shades representing higher proportions. A woody species was considered capable of inhabiting frost zones if it had at least five valid geographical records in these areas, and only ecoregions with twenty or more woody species were included in the analysis.

References:

Karger, D.N., Conrad, O., Bohner, J., Kawohl, T., Kreft, H., Soria-Auza, R.W., Zimmermann, N.E., Linder, H.P., and Kessler, M. (2017). Climatologies at high resolution for the earth's land surface areas. Sci Data 4:170122.

Weigelt, P., König, C., and Kreft, H. (2020). GIFT - A Global Inventory of Floras and Traits for macroecology and biogeography. J Biogeogr 47:16-43.

Wu, W., Guo, L., Yin, L., Cai, B., Li, J., Li, X., Yang, J., Zhou, H., Tao, Z., and Li, Y. (2024). Genomic convergence in terrestrial root plants through tandem duplication in response to soil microbial pressures. Cell Rep 43:114786.

Zanne, A.E., Tank, D.C., Cornwell, W.K., Eastman, J.M., Smith, S.A., FitzJohn, R.G., McGlinn, D.J., O'Meara, B.C., Moles, A.T., and Reich, P.B., et al. (2014). Three keys to the radiation of angiosperms into freezing environments. Nature 506:89-92.

Zomer, R.J., Xu, J., and Trabucco, A. (2022). Version 3 of the Global Aridity Index and Potential Evapotranspiration Database. Sci Data 9:409.
